# Minimal mechanistic component of HbYX-dependent proteasome activation

**DOI:** 10.21203/rs.3.rs-2496767/v1

**Published:** 2023-03-20

**Authors:** Janelle J. Chuah, Tiffany A. Thibaudeau, Matthew S. Rexroad, David M. Smith

**Affiliations:** 1: Department of Biochemistry and Molecular Medicine, West Virginia University School of Medicine, 64 Medical Center Dr., Morgantown, WV USA; 2: Department of Neuroscience, Rockefeller Neuroscience Institute, West Virginia University, Morgantown, West Virginia, USA

## Abstract

The implication of reduced proteasomal function in neurodegenerative diseases combined with numerous studies showing the protective effects of increasing proteasome activity in animal models justify the need to understand how the proteasome is activated for protein degradation. The C-terminal HbYX motif is present on many proteasome binding proteins and functions to tether activators to the 20S core particle. Peptides with a HbYX motif can also autonomously activate 20S gate-opening to allow protein degradation, but the underlying allosteric molecular mechanism is not clear. We designed a HbYX-like dipeptide mimetic that represents only the fundamental components of the HbYX motif to allow rigorous elucidation of the underlying molecular mechanisms of HbYX induced 20S gate-opening in the archaeal and mamalian proteasome. By generating several high-resolution cryo-EM structures (e.g. 1.9Å) we identified multiple proteasome α subunit residues involved in HbYX-dependent activation and the conformational changes involved in gate-opening. In addition, we generated mutants probing these structural findings and identified specific point mutations that strongly activate the proteasome by partially mimicking a HbYX-bound state. These structures resolve 3 novel mechanistic features that are critical for allosteric α subunit conformational changes that ultimately trigger gate-opening: 1) rearrangement of the loop adjacent to K66, 2) inter- and intra- α subunit conformational changes and 3) a pair of IT residues on the α N-terminus in the 20S channel that alternate binding sites to stabilize the open and closed states. All gate-opening mechanisms appear to converge on this “IT switch”. When stimulated by the mimetic, the human 20S can degrade unfolded proteins such as tau, and prevent proteasomal inhibition by toxic soluble oligomers. Collectively, the results presented here provide a mechanistic model of HbYX-dependent 20S gate-opening and offer proof of concept for the robust potential of HbYX-like small molecules to stimulate proteasome function, which could be useful to treat neurodegenerative diseases.

## INTRODUCTION

The proteasome is a key component of the ubiquitin-proteasome system (UPS), responsible for removing damaged or unneeded proteins and regulating major cellular processes^1^. Regulation by proteasome activators (PAs) are critical to ensure that only proper proteins are degraded. Dysregulation of the proteasome has been implicated in several neurodegenerative diseases (NDs), often characterized by impairment of proteasome function^2–7^. In this study, we elucidate a minimal mechanistic model that describes how HbYX(hydrophobic-tyrosine-variable C-terminal residue)-motif-containing PAs activate the core particle of the proteasome. We also designed a small molecule that functionally emulates this mechanism of proteasomal gate-opening and robustly activates the archaeal, yeast, mammalian, and human proteasomes. We also generated a high-resolution cryo-EM structure of the archaeal proteasome in complex with this small molecule activator that elucidates new mechanistic understanding of proteasome activation that is conserved from archaea to humans. Moreover, this proteasome-activating small molecule can reverse inhibition of the proteasome by toxic soluble protein oligomers implicated in neurodegenerative disease, such as amyloid-β, α-synuclein, and huntingtin exon1.

**Figure 1.**
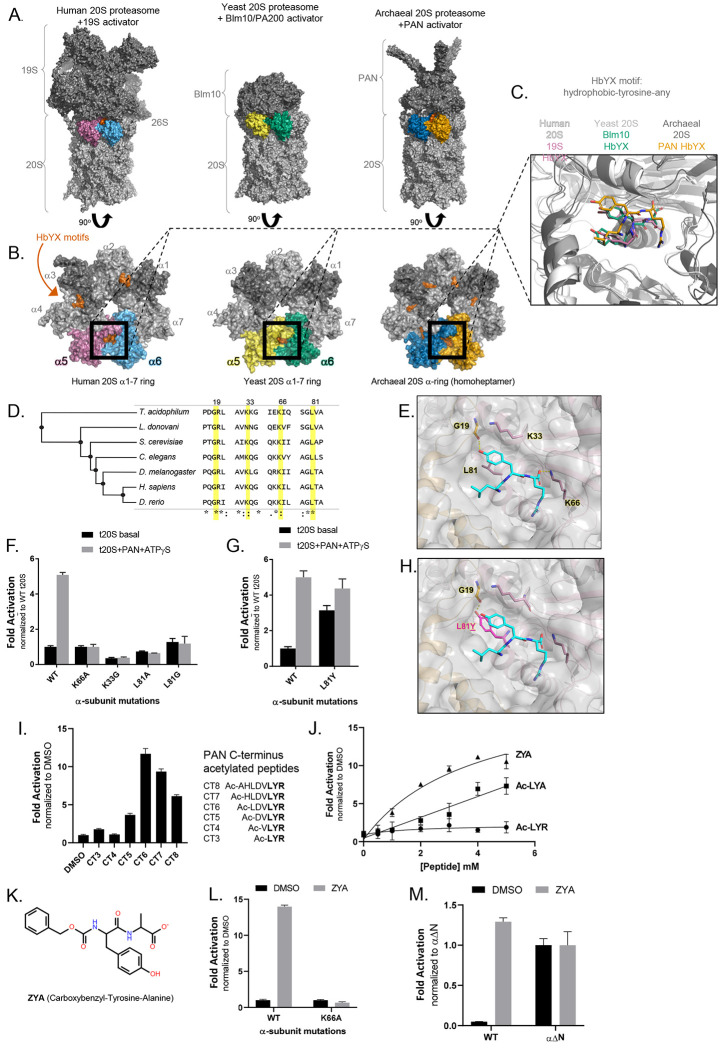
Generation of a minimal HbYX motif mimetic capable of opening the 20S proteasome’s substrate gate. **A.** Surface representation of 20S proteasomes in complex with activators [Human 26S (PDB 6msk), yeast 20S+Blm10 (PDB 4v7o), archaeal 20S + PAN (PDB 6hed)]. HbYX motifs visible are colored red-orange and adjacent α-subunits of the visible HbYX motif are shown in various colors. **B.** Surface representation of the 20S α rings (from A) down the center axis with activator caps removed. Proteasome activator C-termini HbYX residues are shown in red-orange (surface). **C.** Overlay of 20S intersubunit pockets (cartoon) from B with HbYX motif residues (sticks). Crystal structure of PAN C-terminus (PDB 3ipm) is shown in place of Cryo-EM PDB 6hed. Images were rendered with PyMOL. **D.** Multiple sequences alignment of the T20S α subunit with various eukaryotic α6 subunits generated with CLUSTAL OMEGA (1.2.4). **E.** Conserved residues interacting with bound HbYX motif (sticks) in the T20S intersubunit pocket (PDB 3ipm). PAN HbYX motif (LYR) shown in cyan (stick). **F.** Rate of substrate degradation (fluorogenic nonapeptide LFP) by the wild type (WT) T20S proteasome (0.14nM) or K66/K33/L81 mutants incubated with or without PAN (with ATPγS). Stimulation of gate opening was measured by the increase of LFP hydrolysis (rfu/min) relative to WT 20S without PAN. **G.** Experiments with T20S proteasome (0.35nM of wild-type or L81Y mutant) performed same as in F. **H.** Same as (*E*), with L81 mutated to tyrosine (magenta stick). Images in E and H were rendered with PyMOL. **I.** N-terminally acetylated peptides (200 μM) were incubated with 7nM WT T20S proteasomes and LFP (Left). Stimulation of gate opening was measured by the increase of LFP hydrolysis (rfu/min) relative to WT 20S with DMSO. Sequences of peptides are shown (Right). **J.** Dose response of peptides (Ac-LYR, AC-LYA, ZYA) with 7nM WT T20S proteasomes and LFP. LFP degradation rate (rfu/min) is normalized to DMSO. **K.** Structure of ZYA peptide mimetic. **L.** ZYA (2.5mM) incubated with 7nM WT or K66A T20S and LFP. LFP degradation rate (rfu/min) normalized to DMSO. **M.** ZYA (2.5mM) incubated with 7nM WT or gateless (α3ΔN) T2S0 and LFP. LFP degradation rate (rfu/min) normalized to αΔN. Data (means) are representative of three or more independent experiments each performed in triplicate. Error bars represent ± standard deviation.

The core particle of the eukaryotic proteasome, also referred to as 20S (Fig. 1A), consists of four stacked heteroheptameric rings (α-β-β-α) with a central pore for substrate entry. The β rings consist of seven subunits (β1 −7), three of which harbor protease sites. The two α rings also consist of seven subunits (α1-7). Substrate entry in the 20S is regulated by the gate, which primarily consists of the N-terminus of α 2, 3, and 4 extending over the central pore thus closing off this barrel shaped structure^8^. The closed gate conformation blocks the central pore and prevents proteins from entering the 20S to be degraded. The N-terminus of each α subunit carries a YDR (tyrosine-aspartic acid-arginine) motif that interacts with neighboring N-termini to stabilize the closed state of the gate^8^. These N-termini extensions can also change their conformation to an “open” state, whereby they point up and outwards from the α ring pore, which is stabilized by an alternate interaction from the YDR motif^9^. Additionally, truncation of α3s N-terminus (α3ΔN), which act as a central lynchpin to stabilize the closed state, generates a constitutively open (active) 20S that is highly capable of degrading unstructured proteins^8,10^. Assessed by NMR, the basal kinetics of archaeal 20S gate-opening has been suggested to undergo open/close fluctuations on a time scale of seconds^11,12^. Although the kinetics have not yet been measured for the mammalian 20S (M20S), the presence of basal protein or peptide hydrolysis activities suggest that the M20S gate also fluctuates between these states, though likely slower.

While the 20S gate typically favors the closed state, binding of proteasome regulatory complexes (Fig. 1A) to the α ring can trigger conformational changes that cause gate-opening, allowing the 20S to accept unfolded and linearized substrates^13,14^. Two different mechanisms of 20S gate-opening have been described, the HbYX-dependent and the 11S family-dependent mechanisms. Thus far, most studies on the HbYX-dependent mechanism focus on the 19S, also known as PA700 or the Regulatory Particle (RP), which associates with the 20S to form the 26S complex that degrades ubiquitinated proteins (Fig. 1A). The 19S consists of a base subcomplex, which is primarily composed of a heterohexameric ring of ATPases (Rpt1-6), and a lid subcomplex, which contains ubiquitin binding and processing subunits. The 19S has been shown to stimulate gate-opening by the docking of the C-terminal tails of Rpt1-6, some of which contain the HbYX motif, in the intersubunit pockets of the 20S α ring^15^. The use of C-terminal tails to associate with the 20S has also been observed in other Proteasome Activators (PAs) including PAN (proteasome-activating nucleotidase; the archaeal homolog of the 19S), PA200/Blm10 (Fig. 1A, B, &C), and 11S activators.

Not all C-terminal tails of PAs induce gate-opening when bound to the 20S, as evident by the observation that peptides corresponding to the C-terminus of PA26 (a member of the 11S family) cannot induce gate-opening autonomously^16^. Conversely, peptides corresponding to the C-terminus of Rpt2, Rpt3, Rpt5, PAN, and PA200/Blm10 can autonomously induce gate-opening^16,17^. All the peptides that induce gate-opening carry the HbYX motif, which has been shown to be essential for allowing these complexes to associate with the 20S^15,16^. The C-terminal HbYX motif binds into pockets formed by the interface of the α subunits in the 20S, called intersubunit pockets (Fig. 1B&C). Whereas the C-termini of PAN homohexameric ATPases all have a HbYX motif, only the C-terminus of Rpt2, 3, & 5 from the 19S heteromeric ATPases have the HbYX motif and Rpt1 has a partial HbYX motif, lacking the Hb residue. The roles that the C-termini of Rpt4 and Rpt6 (which lack the HbYX motif) play in the association of the 19S-20S and 20S gating regulation are unclear but have been observed bound to intersubunit pockets via cryo-EM^18,19^. The binding of HbYX-peptides to intersubunit pockets, structurally distant from the gating residues, results in gate conformational change. This demonstrates that the HbYX motif functions allosterically, and likely induce substantial conformational changes in the α subunits that in turn, affect the conformation of gating residues^16,20^.

In contrast, the family of 11S activators does not carry a HbYX motif on their C-terminal tails. Their mechanism of gate-opening is relatively well-known compared to the HbYX-dependent mechanism. They associate with the 20S, using their C-termini to dock in the α intersubunit pockets, similar to the HbYX-dependent activators. However, to trigger gate-opening, the 11S family relies on “activation loops” that interface directly with the base of the gating N-termini in the pore of the α ring^21,22^. These activation loops appear to sterically repel a reverse turn proline (Pro17) at the base of the gating residues shifting it by <1 Å, which is sufficient to disrupt the closed state and stabilize the open state^9^. Interestingly, minimal conformational changes in the α subunits (excluding gating regions) are necessary for gate-opening by the 11S activators, as shown by the crystal structure of PA26-20S proteasome^9^. It is evident that the two families of PAs (HbYX-dependent and HbYX-independent) use different strategies to induce 20S gate-opening. Although the location and effect of HbYX-binding has been investigated, the molecular mechanism of HbYX-dependent gate opening appears to be surprisingly complex and remains unsolved.

Structures of the substrate-engaged human 26S (H26S) from Dong et. al ^18^ suggest that the human 19S (H19S) associates with the human 20S (H20S) through multiple interactions between the 19S ATPase’s C-termini and the 20S α-ring. These interactions vary in that they change based on the state of the 26S. As the H26S transition towards a more active state (E_A1,2_ > E_B_ > E_C1,2_ > E_D1,2_)^18^, more C-termini form stable interactions (as observed via cryo-EM), starting with Rpt3, Rpt5 and Rpt2, then Rpt6, and finally Rpt1. The first tails to dock (Rpt3, 5, & 2) all carry the HbYX motif, yet, as visualized by cryoEM, the gate does not appear open. When the last C-terminus of the ATPases binds (Rpt1, which has a partial HbYX motif), a conformational change occurs, resulting in a stably opened gate. Interestingly, another structural study of *Saccharomyces cerevisiae* 26S (Y26S)^23^ suggests the same pattern of C-terminal tail binding for gate opening, while other studies on the Y26S^19,24^ suggest complete gate-opening occurs after the binding of Rpt6, and that the binding of Rpt1 in the α ring is not required for complete gate-opening. Moreover, some Y26S structures^19,24^ indicate that the binding of Rpt2, 3, & 5 alone is sufficient to induce partial gate-opening. Li et. al. previously demonstrated via a biochemical study that the M20S gate was “variably modulated” when complexed with the 19S and could exist in various states of openness. While cryo-EM findings can approximate gate openness, the structures do not precisely reflect the dynamics of the gate within an individual proteasomal state (E_A1,2_, E_B_, E_C1,2_, E_D1,2_). For example, the un-activated 20S proteasome by itself still can degrade linearized proteins or peptides, but structurally, it is observed with a closed gate via cryo-EM or X-ray crystallography. Therefore, structural methods have a limited ability to draw conclusions regarding the functional openness of a gate.

The reported disparities between the HbYX-dependent mechanism of gate opening in the H26S and Y26S raises the question of how, if so, HbYX-motif binding opens the gate. It is possible that HbYX-dependent gate opening requires occupation of a minimum number of intersubunit pockets to trigger an allosteric transition. Alternatively, it is possible that only a subset of pocket(s) must be occupied by the corresponding C-termini to trigger gate-opening, or some combination of these two scenarios. We expect that since the HbYX motif is conserved from archaea to humans and is conserved in most PA’s (excluding the 11S family), the HbYX-dependent mechanism of gate-opening would also be conserved.

Recently, we showed that ND-associated proteins (i.e., amyloid-β, α-synuclein, and huntingtin) can fold into a common conformation that inhibits 20S and 26S proteasomes^20^. We also learned that these soluble oligomers (A11+) inhibit the 20S by allosterically stabilizing the closed gate conformation^20^. This negative allosteric regulation by such toxic^25^ oligomers appear to be mechanistically coupled to the HbYX-dependent mechanism of 20S gate-opening^20^, suggesting that these oligomers and the HbYX motif are allosteric regulators of the same gating mechanism. Therefore, we hypothesize stimulating 20S activity via the HbYX-dependent mechanism will antagonize impairment by ND related oligomers, which could restore proteasome function and stimulate protein degradation, thereby potentially providing a therapeutic approach for ND. While the molecular interactions that stabilize the closed and fully open state of the proteasome’s gate are well-studied^8,26^, as are the 26S proteasome opened/closed states^18,19,23,24^, the complex molecular mechanisms that allosterically regulate the transition between these closed and open states are not understood. A clear understanding of the gate-opening mechanism in the 20S will provide the molecular framework to guide drug-discovery approaches aimed at activating proteasomal degradation to treat ND and is expected to help elucidate how ND-associated oligomers impair proteasome function.

## RESULTS

### Identifying essential structures for HbYX dependent gate-opening.

The HbYX motif is highly conserved and found in archaeal, yeast, and human proteasome activators, among other species (Fig. 1A, B, & C). Through mutagenesis and structural analyses, we found that the intersubunit pockets contain multiple conserved residues, some of which are already known to be important for gate activation (i.e., Pro17, Lys66 from *Thermoplasma acidophilum*)^9,16,27^ (Fig. 1D). To better understand how the HbYX motif interacts with the 20S, we aligned HbYX-bound intersubunit α pockets from human (PDB 6msk, cryo-EM), yeast (PDB 4v7o, crystallography), and archaeal proteasomes (PDB 6hed, cryo-EM) (Fig. 1C). We observed in these structures that the HbYX motif from these three diverse species binds to the 20S intersubunit pockets in highly similar orientations, with three distinct and shared interactions (Fig. 1D&E): (1) The C-terminal carboxylic acid of the motif is directed towards the conserved lysine (K66 in archaea), which is already known to be required for 20S proteasome-activator complex formation, (2) the penultimate tyrosine’s hydroxyl group hydrogen-bonds with the backbone carbonyl of G19 and is oriented toward the proline reverse turn (located at the base of the gate), and (3) the hydrophobic (Hb) group contacts a hydrophobic pocket in the neighboring α subunit.

We investigated the effect of mutating three of the conserved residues that are positioned to interact with the HbYX-motif (Fig. 1F) in the *T. acidophilum* 20S (T20S). We chose the T20S because it is a symmetric homoheptamer, which facilitates simultaneous mutagenesis in all 7 intersubunit pockets. We then tested activation of the T20S mutants by PAN (measuring LFP nonapeptide degradation) since PAN uses the HbYX-dependent mechanism (Fig. 1F). Consistent with previously published results^16^, K66A mutation prevented PAN activation of the T20S. The basal activity of T20S-K66A was similar to the wild type (WT). Structural studies showed that the aliphatic portion of the K33 side chain and L81 are positioned on either side of the HbYX tyrosine ring, interacting hydrophobically to hold the HbYX tyrosine ring in place (Fig 1E). When K33 was mutated to glycine, the mutant T20S could no longer be stimulated by PAN (Fig. 1F). Also, T20S-K33G basal activity was about half of the wild-type. Similarly, the mutation, L81A (located directly under the HbYX tyrosine) prevented PAN-mediated proteasome activation. Therefore, based on prior structures and these biochemical results, we conclude that K33 and L81 are both important for stabilizing and, likely, orienting the HbYX tyrosine in the intersubunit pocket.

Considering the importance of HbYX tyrosine hydrogen-bonding with the backbone carbonyl of G19, we investigated whether substituting tyrosine for L81 (L81Y) could mimic the effect of a bound HbYX motif, as the mutation would place a tyrosine ring in similar space as the HbYX tyrosine (Fig. 1H). We found that proteasomes with the L81Y mutation (T20S-αL81Y) had elevated basal activity, relative to WT (Fig. 1G), suggesting that the mere placement of a tyrosine into this location is sufficient to partially induce gate opening, similar to HbYX binding. In agreement with this conclusion, addition of PAN at saturating concentration to T20S-αL81Y proteasomes resulted in a further 30% increase in activation to near WT-like activation (Fig. 1G), indicating the L81Y gate was not completely opened by mutation alone. These results demonstrate that the proper placement of a tyrosine alone in all intersubunit pockets of the archaeal 20S proteasome is sufficient to induce conformational changes leading to at least partial 20S gate opening.

### Generating a minimal HbYX motif-based peptide to stimulate gate opening.

Since the L81Y mutation was able to induce partial gate opening, we hypothesized that small peptides mimicking the HbYX-motif could activate 20S gate opening. Our previous study in 2007 showed that only peptides >7 residues long with a C-terminal HbYX motif (corresponding to the PAN’s C-terminus) could induce gate opening^16^. However, this was determined with peptides that had unmodified N-termini. We suspected that the charged N-terminus may prevent shorter HbYX peptides from binding to the intersubunit α pockets. To test this hypothesis, we synthesized the same peptides corresponding to the PAN C-terminus from three to eight residues in length (CT3-8), except with an acetylated N-terminus. N-acetylated PAN C-terminus peptides shorter than seven residues activated the 20S (Fig. 1I). Notably, we observed that N-acetylated PAN C-terminus peptide six residues in length (CT6) activated the 20S over 10-fold greater than the control; even the 3-residue peptide (CT3) had (~2-fold) gate-opening activity (Fig 1I).

Based on the successful gate-activation of PAN CT3 (Ac-LYR) after a single modification (acetylated N-terminus), we investigated whether additional modifications would further improve the efficacy of the peptide. Our previous study showed that the substitution of alanine for arginine does not affect PAN-CT8 peptide efficacy to bind α intersubunit pockets and open the gate^16^. We therefore substituted an alanine in place of the bulky arginine residue, which resulted in greater activating efficacy, compared to Ac-LYR (Fig. 1J). Attempting to further minimize the size of the molecule, we chose carboxybenzyl (Z) as an N-terminal blocking group that could eliminate the N-terminal charge and mimic the Hb group (of the HbYX motif). Combining these modifications resulted in a dipeptide with a hydrophobic group preceding the N-terminus of tyrosine (Z-Tyr-Ala or ZYA) (Fig. 1K). We compared ZYA against Ac-LYR and Ac-LYA and observed significant improvements in T20S activation (Fig. 1J). ZYA could substantially activate T20S activity, but its affinity was poor (see below). While ZYA could activate T20S ~13 fold, it could not stimulate gate-opening in T20S-K66A to any extent (Fig. 1L), demonstrating that ZYA requires K66 in the intersubunit pocket as expected. This suggests that activation of T20S by ZYA occurs through interactions similar to those responsible for activation by PAN. To rule out the possibility that ZYA might be activating the protease sites rather than inducing gate opening, we also tested the capacity of ZYA to stimulate the αΔN-T20S, which lacks gate-residues and is constitutively open. ZYA did not stimulate the activity of αΔN-T20S to any extent (Fig. 1M). Together, these results demonstrate that ZYA activates the 20S by inducing gate-opening, similar to the HbYX motif on which it was based.

### Elucidating ZYA’s mechanism of activation using cryo-EM

**Figure 2:**
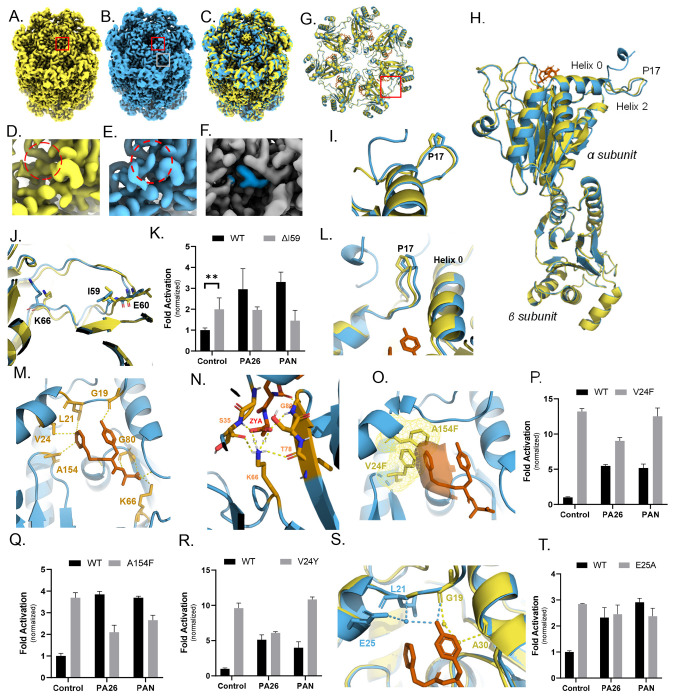
Structure of ZYA bound to the T20S proteasome (1.9Å) and supporting mutations demonstrating the mechanism of HbYX induced gate opening. **A.** WT T20S unsharpened electron density map (yellow; 2.06Å); red square highlights the view in D. **B.** T20S-ZYA unsharpened electron density map (blue; 1.9 Å); red square highlights the view in E; gray square highlights the view in F. **C.** Overlay of WT T20S (yellow) and T20S-ZYA (blue) unsharpened electron density maps showing clear conformational differences in the maps. **D.** WT T20S electron density map (yellow) of boxed region in A, with red dotted circle highlighting location of the YDR motifs tyrosine in the open state. Absence of density in this location indicates a “closed” state, which is confirmed by densities, at lower map threshold levels, near the center of the α ring (seen in A). **E.** T20S-ZYA electron density map (blue) of red box region in B with red dotted circle highlighting density corresponding to tyrosine of YDR motif in the gate open state. **F.** T20S-ZYA electron density map (gray) of α intersubunit pocket (grey box region in B), with densities corresponding to bound ZYA ligand colored blue. **G.** Top view of the atomic model of WT-T20S (yellow) and T20S-ZYA (blue) overlayed after alignment of β subunit rings. Showing only the α subunits here for clarity. **H.** Overlay of WT (yellow) and T20S-ZYA (blue) α and β subunits models, aligned by β subunit. **I.** View of Helix 0 of alignments from H with key residue Pro17 shown in sticks. The single α subunits are aligned to show intrasubunit conformational changes in Pro17. **J.** View of loop proximal to K66 from G red box, with key residues discussed shown in sticks, aligned by α subunit to demonstrate intrasubunit conformational changes. Similar changes are seen in β alignments. **K.** 7nM T20S (WT or ΔI59) incubated with 44nM PA26 or 15nM PAN (supplemented with ATP and MgCl_2_). LFP degradation rate (rfu/min) normalized to control and the amount of proteasome from different preparations is normalized to their rate of LLVY-AMC hydrolysis, which is insensitive to gating affects (see methods for details). **L.** Close-up on Helix 0 and Pro17, shown in sticks, aligned by β subunit to demonstrate intersubunit conformational changes. **M.** ZYA (red) in the α intersubunit pocket, showing key interactions with α subunit residues (orange). **N.** A network of ionic interactions between ZYA’s C-terminal carboxy, K66 side chain, and indicated residues in the α-intersubunit pockets that stabilize the shifted state of K66 that can be seen in J. **O.** Structure of T20S-ZYA with simulated mutation of A154F and V24F shown in yellow sticks with dotted clouds, showing overlap between these mutants and the Z group binding location (ZYA). **P.** 7nM T20S (WT or V24F) incubated with 44nM PA26 or 15nM PAN (supplemented with ATP and MgCl_2_). LFP degradation rate (rfu/min) normalized as in K. **Q.** Same as N but 7nM T20S (WT or A154F). LFP degradation rate (rfu/min) normalized as in K **R.** Same as N but 7nM T20S (WT or V24Y). LFP degradation rate (rfu/min) normalized as in K. **S.** Model of ZYA (red) docked in α intersubunit pocket, aligned by β subunits, showing interactions with waters (spheres) and residues (sticks). **T.** Same as N but 7nM T20S (WT or E25A). LFP degradation rate (rfu/min) normalized as in K. *Data (means) are representative of three or more independent experiments each performed in triplicate. Error bars represent ± standard deviation for panels K, N, O, Q and S.

Using cryo-EM, we generated a 1.9Å structure of ZYA bound to T20S (ZYA-T20S) (SFig. 1&2) (Fig. 2B) and a 2.1Å WT T20S structure (Fig. 2A) (SFig. 3&4). We noted strong densities in our ZYA-T20S map, corresponding to the YDR region (Fig. 2B&E) in a conformation that corresponds to the open-gate state (Fig. 2C), with the N-termini pointing up and a lack of density in the central channel. This structure also resolves the N-termini of the α subunits up to Gly4, which include three N-terminal residues that have not been previously resolved. The WT T20S map, as expected, does not contain these open state densities but does show clear pore-central densities that are expected for the closed gate (Fig. 2A&D). In addition, our new WT map also resolves the N-termini up to Ala11, which includes 2 additional residues not previously resolved in the closed state. In the ZYA-T20S map, densities corresponding to ZYA bound to the α intersubunit pockets were clearly visible (Fig. 2F) and resembled the expected structure of this dipeptide. To determine the intersubunit (around the ring) and intrasubunit (within a single subunit) conformational changes induced by the binding of ZYA, we compared the ZYA-T20S structure to our 2.1Å structure of the WT T20S (PDB:) (Fig. 2C, G, H; SMovie 1). For the intersubunit changes we first aligned the subunits in the β-ring (Fig. 2H), to allow for visualization of changes in the α ring (no significant changes were seen in the β subunits). These intersubunit conformation changes are presented as a rotation of the α-ring (SMovie 1). This apparent rotation in the α-ring is primarily due to individual rigid body movements of each α-subunit (Fig. 2C&G), centered around the length of Helix 2 which acts as the pivot. Helix’s 3, 4, and 5, which are most distant from the pivot move by ~2Å.

In contrast, intrasubunit conformational changes were assessed by aligning a single α subunit from WT T20S and T20S ZYA structures. Though subtle, these intrasubunit changes were present. The Pro17 loop and connected N-terminal extension were shifted by ~1.0Å in a direction perpendicular to Helix 0 (Fig. 2I), for comparison, the intersubunit change of Pro17 is 1.3Å so most of the Pro17 shift comes from intrasubunit changes. Even Helix 0 shifted by about ~0.6Å (1.0A for intersubunit changes) moving in the direction pointed towards the Pro17 loop (Fig. 2I). These subtle intersubunit changes were also clearly visible in the electron density map (Fig 2C). In addition, intersubunit conformational change was observed in the loop (S50-E65; back-loop), which is in the outer portion of the intersubunit pocket, adjacent to K66 (Fig. 2J). Though the local resolution of this loop is around 2.7Å, it clearly changes conformation, with the bottom of the loop from I59 to K66 moving in the direction of K66 anywhere from ~1-2Å. This motion appears to be causing or accommodating the rotation of the α subunits described above. We conclude that binding of the HbYX-like ZYA molecule causes unique inter- and intra-subunit conformational changes that allosterically switch the T20S from the closed to the open state.

Next, we analyzed the HbYX dipeptide’s interactions with the intersubunit pocket to deduce its mechanism for activating proteasome gate-opening (Fig. 2M). The carboxybenzyl group (i.e., Hb group) docks in a hydrophobic pocket, interacting with V24, L21, and A154, 3.7Å, 4.3 Å and 3.6Å away, respectively (Fig. 2M). The hydroxyl group of ZYA’s tyrosine hydrogen bonds with the backbone of G19 (Fig. 2M), sandwiched between L81 and K33, as would be expected for the penultimate tyrosine of the HbYX motif^15,28,29^. Additionally, we noted the backbone of the dipeptide hydrogen bonding with the backbone of G80, V82, and ZYA’s C-terminal carboxylic group forming a salt bridge with the sidechain of K66 (Fig. 2M), consistent with previous observations on the importance of K66 for HbYX-dependent gate-opening^9,16,27^. These noted interactions indicate that ZYA binds essentially identically as has been shown for other HbYX motifs (compare Fig 2M to 1C).

In addition to the above-mentioned ionic interactions, we also noticed the K66 and ZYA carboxy group participate in a highly coordinated network of H-bonds due to the new position that K66 takes after ZYA binding. In fact, in addition to the K66 interaction the carboxy group of ZYA also interacts with the backbone of G80 and S35, flanking both sides of the carboxy-K66 salt bridge (Fig. 2N). Moreover, Lys66 is also H-bonds to the backbone of S35 and T78, also flanking both sides of the salt bridge. It appears that this network of 6 distinct ionic interaction stabilizes the K66 in this new position, likely stabilizing the rearrangement of the K66 adjacent back loop.

The high resolution of these structures also allows us to model waters into the T20S. We therefore analyzed how the binding of ZYA might affect water molecules in the intersubunit pockets, that could potentially affect the conformation of the α subunits and the gate. Based on the modeled waters, we found that ZYA’s tyrosine displaced a water molecule that hydrogen bonds with the backbone of A30 and another water that further hydrogen bonds with G19 (Fig. 2S). Concurrently, we noted a new water positioned to hydrogen bond with the hydroxyl group of tyrosine, and the backbone nitrogen of L21 and the side chain of E25, residues belonging to the neighboring α subunit Helix 0. These water molecules are well positioned to potentially be important for stabilizing the different states of the T20S gate (more below).

The ZYA-T20S model also showed that the binding of ZYA to the intersubunit pocket shortens the distance between G19 on one subunit and K66 on the neighboring subunit by ~1Å (essentially the walls of the intersubunit pocket are pulled together in the range of 1-2Å), primarily due to a shift of K66 α carbon (Fig 2J&M). Based on these interactions and changes in the pocket, we deduce that ZYA binding acts as a “cable” that bridges across the intersubunit pocket connecting Helix 0 of an α subunit to the K66 loop in the neighboring subunit. The length of this “cable” is just short enough to “pull” or shift the K66 towards its new position (Fig 2J and N). The result of displacing this K66 appears to be a rearrangement of the adjacent back-loop, resulting in the end of the loop (e.g., L57) moving in a direction away from the neighboring subunit and towards K66 by ~1.2Å. In addition, the neighboring subunit Helix 3 follows the K66 loop to cause or accommodate the rigid body rotation of the α subunit (SMovie 1). Thus, the intersubunit “bridging” role ZYA plays leads to a K66 shift that promotes a rigid body rotation of the α subunits. Interestingly, the rigid body rotation and intrasubunit shifts in Helix 0 combine to shift Helix 0 away from the Pro17 that is in the neighboring subunit (Fig 2L and SMovie 1). Since the base of Pro17 packs against Helix 0 in the neighbor, and since Pro17 is on a flexible loop, it is able to move with its neighboring Helix 0 causing it to shift ~1.1Å, which is known to be associated with gate-opening. We further tested these conformations induced by ZYA binding and new mechanistic insights via mutagenesis.

To further elucidate ZYA’s mechanism of activation, we mutated multiple residues to emulate or perturb the conformational changes previously discussed and observed their effect on gate-opening by HbYX-dependent and -independent mechanisms. ZYA binding shifted the back-loop proximal to K66 (Fig. 2J) towards K66, so we asked whether shortening the loop by a single residue deletion affect gating. The deletion of I59 (ΔI59) (Fig. 2K) resulted in slightly higher 20S activity (p-value: 0.0013), e.g., a more open gate (Fig. 2K). In addition, neither PAN (a HbYX-dependent activator) nor PA26 (non-HbYX-dependent activator) could stimulate the activity of T20S-ΔI59 (Fig. 2K). This mutation suggests that the loop proximal to K66 does affect gating as expected, demonstrating that this K66 back-loop is important for regulating gate-function.

Next, we asked what role the Hb (i.e., Z) group on ZYA plays in gate opening, does it contribute to affinity, or does it actively play a role in inducing gate opening. To ask these questions, we mutated V24 and A154 (both in the Hb binding pocket) to phenylalanine, emulating the binding of the Z (benzene) in the pocket. Mutagenesis of these two residues to phenylalanine in Pymol shows they would occupy overlapping space with the Z group of ZYA (Fig 2O). Both T20S variants, V24F and A154F did in fact have a far higher activity than the WT control (Fig. 2P&Q), with V24F being the most activating (~14fold). Additionally, PAN and P26 could neither further stimulate V24F and A154F mutations (Fig. 2P&Q). This could be because the gate could not be further opened, or it could be that altering the Hb binding sites prevents them from binding to the 20S. Since V24F stimulated gate opening so strongly, even stronger than WT plus saturating PA26, this suggests that introducing a large aromatic group in the Hb binding pocket by itself is sufficient to cause maximal gate opening. To test this hypothesis, we generated a V24Y variant, mutating V24 to another residue with a large aromatic group that is also observed to be in the Hb position of the HbYX motif on some proteasome activators (e.g., human Rpt5, yeast Blm10, mammalian PA200). As hypothesized, V24Y T20S had higher activity than the WT control and PAN and PA26 could not further stimulate (Fig. 2R). These indicate that the Z group of ZYA likely plays an important and direct role in ZYA’s mechanism of action.

Recognizing the possibility of water being involved in ZYA mechanism of activation, we sought to further elucidate how they contribute to the conformation we observed. We mutated E25, a residue described above to interact with a water molecule that further interacts with L21 and ZYA’s tyrosine. If E25’s role in interacting with waters is critical, we predict that E25A would perturb ZYA activation. Surprisingly, E25A T20S exhibits higher basal activity compared to WT T20S (Fig. 2T) indicating E25 helps stabilize the closed state. More importantly, neither PAN nor PA26 could activate E25A T20S. This suggests that the E25’s ability to localize a specific water may be important for either regulator binding or the switching to the gate-open state. These results suggest that hydration of the intersubunit pocket, or at least E25 may play important roles in 20S gating. However, the confidence of accurately identifying water molecules using cryo-EM, e.g., compared to crystallography, is not high (even at 1.9Å), and thus other explanations could always be possible. Despite this caveat, we did consistently see the same water densities in different structures presented here (see below), and their expected displacement by ligand binding.

**Figure 3:**
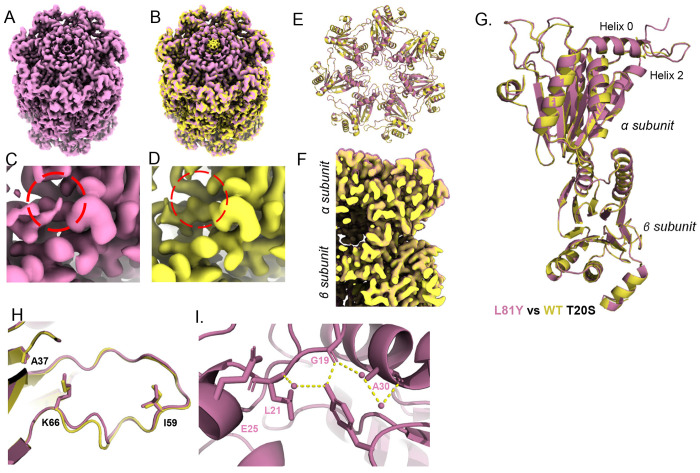
Cryo-EM structure of T20S-L81Y (2.4Å) mutant demonstrates partially open state by moving helix 0 without inducing HbYX-like rotations of the α subunits. **A.** T20S-αL81Y unsharpened electron density map (pink). **B.** Overlay of WT T20S (yellow) and T20S-αL81Y (pink) unsharpened electron density maps. **C.** T20S-αL81Y electron density map (pink) of partial α ring (top view) with red dotted circle highlighting density corresponding to the position of the tyrosine of YDR motif in the open state. **D.** WT T20S electron density map (yellow) of partial α ring (top view) with red dotted circle highlighting missing density corresponding to tyrosine of YDR motif. **E.** Top view of atomic models showing overlay of α subunits of WT (yellow) and T20S-αL81Y (pink), aligned by β subunits. **F.** Cross section of overlayed electron density maps from WT T20S (yellow-solid surface) and T20S-αL81Y (pink mesh surface). **G.** Overlay of α and β subunit from atomic models of WT (yellow) and T20S-αL81Y (pink), aligned by β subunits. **H.** Close-up on loop proximal to K66, with key residues discussed shown in sticks. Coloring as in E. **I.** Model of T20S-αL81Y showing interactions between L81Y with waters (spheres) and labeled residues (sticks).

Realizing the pivotal role tyrosine played in our ZYA-T20S structure, and the partial activation induced by the penultimate tyrosine mimicking L81Y mutation (Fig. 1G), we generated a cryo-EM structure of T20S-αL81Y (2.3Å) to more rigorously evaluate how tyrosine contributes to gate-opening (SFig. 5&6) (Fig. 3A). Our EM map indicated expected densities corresponding to the open state of the YDR region in our map (Fig. 3C-red circle), which were not visible in our WT T20S map (Fig. 3D). However, compared to the ZYA-T20S map which we presumed to have a fully opened gate, the YDR densities in the L81Y map were weaker (compare Fig. 2E to 3C), suggesting a partially opened gate, as previously indicated by our biochemical data (Fig. 1G).

To determine conformational changes induced by the single mutation mimicking the penultimate tyrosine, we compared the T20S-αL81Y model against our WT T20S (Fig. 3B, E, F & G; SMovie 2) and ZYA-T20S models. Unlike the ZYA-T20S model, the conformational changes caused by L81Y were different and subtle. We did not observe a substantial α-ring rotation or a conformational change at the loop proximal to K66 (Fig. 3B, E, &H); however, we did observe a slight rise of Helix 0 in a direction parallel with the 7-fold axis mostly clearly seen in Fig 3B. In fact, we noted a slight rise of the entire surface of the α subunits away from the β ring, with the most prominent changes in Helix 0 (Fig. 3B, E and F). Interestingly, alignment of individual α subunits revealed minimal intrasubunit conformational changes at this resolution. We presume the subtler effect is due to the slightly different placement of tyrosine in the L81 position compared to the tyrosine in ZYA when bound to the T20S (compared Fig. 2M to 3I). Similar to ZYA-T20S, the L81Y mutation caused minimal or no conformational changes in the β subunits (Fig 3F&G).

The T20S-αL81Y model shows that the hydroxyl group of tyrosine is within proximity to hydrogen bond with the backbone of G19, similar to ZYA’s tyrosine. Additionally, we noted that tyrosine hydrogen bonded with a water molecule that is also hydrogen bonding to the backbone of L21 but not the side chain of E25 (Fig. 3I). We also noted the water which hydrogen bonds with the backbone of A30 is not displaced by L81Y, reinforcing the point that L81Y is not oriented like ZYA’s tyrosine. Collectively, our structure suggests that the interactions with G19 and L21 are sufficient to cause a Helix 0 shift upward, which consequently at least partially opens the gate. Therefore, the L81Y tyrosine by itself cannot fully mimic the ZYA bound open state. This indicates that the bridging effect between G19 and K66 implemented by ZYA is likely important to induce the α subunit rotation that leads to the fully open state. Insight into how the L81Y partially induces gate opening will be further discussed below.

### Conformational changes in the N-terminus of these activated T20S structures uncover new interactions that stabilize the open and closed gate conformations

While analyzing our structures to identify interactions responsible for stabilizing the gate in the open state, we noted conformational changes on the N-terminal tail in both ZYA-T20S and T20S-αL81Y models. The structure of the N-terminal tail in the closed gate conformation has not been well resolved in previously published structures of the T20S proteasome; thus, our high-resolution structures of the WT T20S (2.1Å), ZYA-T20S (1.9Å), and T20S-αL81Y (2.3Å) provide novel insight to how the gate is stabilized in the close and open states.

**Figure 4:**
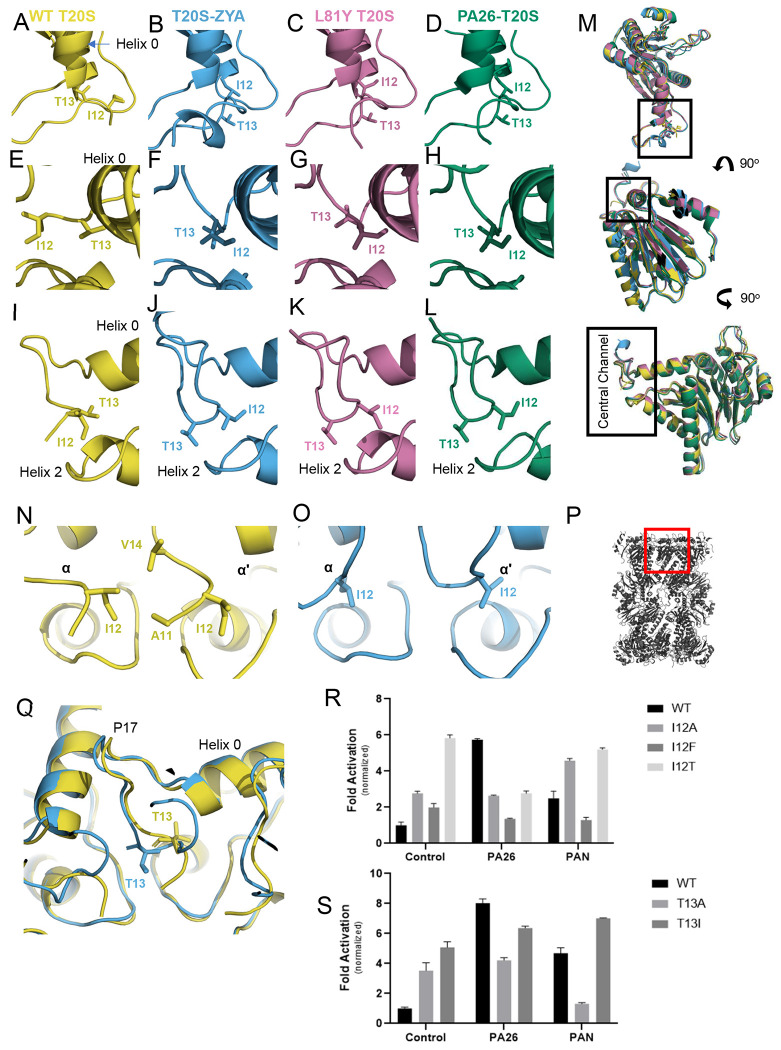
High-resolution WT-T20S structure (2.1Å) combine with ZYA-T20S structure shows I12-T13 play pivotal mechanistic role in switching the gate between open and closed states. **A.** View of WT T20S (yellow) IT switch residues, I12 and T13 (sticks) in the closed gate state. **E.** Same as A but rotated 90°. **I.** Same as E but rotated 45°. **B,F&J** | **C,G&K** | **D,H&L**. Same as A,E&I but for ZYA-T20S (blue), T20S-αL81Y (pink) and PA26-T20S (green), respectively. **M.** (Top) View of IT Switch (box) on a single α subunit, corresponding to A, B, C and D; (middle) same as top after being rotated 90 degrees as shown, corresponding to E, F, G, and H; (bottom) same as middle after being rotated 90 degrees as shown, corresponding to I, J, K, and L. **N.** I12 (left residue) of the IT switch in proximity to V14 and A10 (sticks) from neighboring α subunit in the WT-T20S, corresponding to closed gate state. **O.** Same view as I, except in ZYA-T20S in the open state. I12 does not interact with neighboring α residues but instead interacts under helix 0. **P.** Lengthwise cross section view of 20S, oriented as shown in N and O. **Q.** Overlay of WT T20S and T20S-ZYA showing the IT switch in the closed (WT-T20S, yellow) and open (ZYA-T20S, blue) states. **R.** 7nM T20S (WT, I12A, I12F, or I12T) incubated with 44nM PA26 or 15nM PAN (supplemented with ATP and MgCl_2_). LFP degradation rate (rfu/min) normalized as in fig. 2K). Data (means) are representative of three or more independent experiments each performed in triplicate. Error bars represent α standard deviation. **S.** 7nM T20S (WT, T13A, or T13I) incubated with 44nM PA26 or 15nM PAN (supplemented with ATP and MgCl_2_). LFP degradation rate (rfu/min) normalized as in Fig. 2K. Data (means) are representative of three or more independent experiments each performed in triplicate. Error bars represent ± standard deviation.

Our new cryoEM structure of the WT T20S now resolves more of the N-termini in the closed state, showing clear density beyond the prior resolved T13 to also show the location of I12 and A11. In this new T20S model, the T13 side chain occupies the space between Helices 0 and 2 (Fig. 4A, E, and I) and I12 is seen binding to a hydrophobic pocket created by the neighboring subunits N-termini comprising A11, I12 and V14 (Fig. 4N). Interestingly, the ZYA-T20S model shows that T13 is pulled out of the pocket from under Helices 0 and now I12 binds into this same pocket, which is mostly hydrophobic, containing A11, I12, and V14 (Fig. 4 A versus B, E versus F, I versus J; SMovie 3). Simply put, I12 and T13 switch binding locations under Helix 0 to switch from the closed to the open state. It appears that the ZYA-binding induced rotation of the α-subunit, that is associated with the movement of Helix 0 and displacement of P17, “pulls” T13 out of the Helix 0 pocket, and I12 closer to this pocket, allowing it to bind in this position to stabilize gate opening (Fig 4Q and SMovie 3). Going forward, we refer to the I12, T13 motif and this switching mechanism as the “IT switch”.

We next looked at I12/T13 in the structure of the T20S-αL81Y and found that it was similar to the ZYA-bound state (compare Fig. 4C, G, K to Fig 4B, F, J; SMovie 4) indicating that mutation of L81Y also induced gate-opening via the IT switch. However, as expected, the T20S-αL81Y EM density corresponding to the two residues were not as well resolved compared to the map of ZYA-T20S (see density fit in SFig. 7A), since the resolution of this structure was not as high, and this mutant did not generate as strongly open state as ZYA. The L81Y’s effect on the IT switch is interesting, since there is minimal displacement of Pro17, and the only significant conformational change is the rise in Helix 0. Based on this we hypothesize that the rise in Helix 0 slightly alters the IT switch binding pocket, making it more favorable to bind I12 over T13. Thus, pushing the structural equilibrium toward the open state by primarily only affecting the IT switch binding pocket. Mutation of the IT switch presented below lends further support to the effects on the IT switch binding pocket.

To elucidate if the IT switch is specific to the HbYX-dependent gate opening, we compared our new WT-T20S model to the previously published PA26-T20S model (PDB:1YA7), which could provide new insights. Interestingly, we observe a similar conformational change in the IT switch of the PA26-T20S structure (Fig. 4D, H, & L; SMovie 5). It is apparent that the displacement of Pro17 allosterically triggers switching of the “IT switch” and that the PA26-induced open conformation of the IT switch specifically is essentially identical to the ZYA bound state. The IT switch mechanism functioning to stabilize gate opening by ZYA-binding, mutation of L81Y and PA26-binding demonstrates that the IT switch is a general structural feature of the open and closed states of the T20S. However, all three mechanisms of activating the IT switch are implemented in mechanistically distinct ways, each of which affects the IT switch.

To further confirm that the IT switch plays a central role in proteasome gating, we mutated the I12 and T13 residues. Both I12 and T13 appear to play roles in both the open and closed structures. For example, in the closed state I12 is bound to the neighboring N-termini’s hydrophobic pocket (Fig 4N), and T13 is bound under Helix 0; then in the open state I12 binds under Helix 0 and T13 binds near V129/R130 in the other neighboring subunit (Fig 4Q). These dual roles in both states complicate effects due to mutagenesis so we first asked what the effects would be on the basal WT T20S activity. Importantly, disruption of the closed conformation could have two different effects: 1) stabilization of the open state or 2) an increase in structural disorder/entropy of the N-termini which could have a partial activation effect. Prior studies suggest the N-termini of the closed state in the T20S are disordered, but our new WT structure shows a high degree of order up to residue 11 with residues 1-10 being disordered, though the extent of this disorder is not known.

We first mutated 112 to alanine (A), phenylalanine (F) or threonine (T) (Fig. 4R). The I12A mutant is expected to reduce hydrophobic interactions with the neighboring subunits A11/V14 pocket in the closed state, and indeed this mutant was about 3-fold more active than WT (Fig. 4R), which is consistent with 112’s role stabilizing the closed state. The I12F had less of an effect with ~2-fold activation (Fig. 4R), which is consistent with more retention of hydrophobic interaction with its neighbor despite the added mass. The I12T mutation showed 6-fold activation (Fig. 4R), consistent with this polar residue not being supportive of the required hydrophobic interactions with the neighbors A11 and V14 residues. We next mutated the T13 residue to alanine or isoleucine (Fig. 4S). The T13A mutation again increased the basal activity of this mutant (~3.5 fold) (Fig. 4S), which would be expected with a loss of interactions with the IT switch pocket under Helix 0 due to substitution with the much smaller alanine sidechain. Similarly, the T13I mutation resulted in ~5-fold activation (Fig. 4S). This was unexpected since isoleucine can bind to the IT switch pocket, in both the ZYA and PA26 induced open states. We hypothesize that the T13I mutation increases the interaction of the isoleucine with the neighboring V129/R130 binding pocket in the open state, rather than disrupt the closed state. Regardless, these results demonstrate that these IT switch residues are important for the maintenance of the closed state of the proteasome and its overall function.

We next determined if these IT switch mutants could be activated by either PA26 or PAN. Interestingly PA26 could not activate any of the 112 mutants we generated compared to the control, and even reduced the activity of the I12T mutant. Likewise, PAN could not activate the I12F or I12T mutants but did activate the I12A by almost 2-fold. First these results again demonstrate the critical function of the IT switch in regulating gate-opening by these proteasome regulators. Furthermore, since PAN can activate I12A, but PA26 cannot, this suggests that the HbYX and PA26 mechanisms are indeed distinct. It appears that the α-subunit rotation induced by HbYX binding alters the IT switch pocket (different than PA26 does), allowing some compatibility with the I12A residue docking under Helix 0 to stabilize the open state to some extent. Since the I12F mutation showed the least activation of the T20S, and completely prevented PA26 or PAN from stimulating gate-opening, we interpret this to indicate that the phenylamine side chain is to large and bulky to properly fit in the IT switch binding pocket, and thus prevents stabilization of the open state. The I12T mutation, however, is very activating and stimulates gate opening as well as PA26 binding does. Thus, the fact that PA26 nor PAN can further activate this mutant is not very surprising since it may be fully open in the basal state. The inhibition of this mutant by PA26 but not PAN suggests again that these regulators affect gating mechanistically in different ways. For the T13 mutants, PA26 did not stimulate T13A (p-value: 0.082) and slightly stimulated T13I, but not to the level of WT. PAN, however, significantly inhibited T13A and stimulated T13I a bit more than PA26. Thus, it would appear that the T13I does not form a stable closed structure (discussed above) and can only be stimulated to a more open state by a small amount. The T13A mutation, however, again shows different results for these two activators, with PA26 having minor impact on its activity by PAN substantially inhibiting it. This again suggests distinct roles of PAN and PA26 in how they induce gate opening. Together these data strongly support the role of the IT switch in being a central player in regulating the closed and open states of the proteasome gate.

### Conservation of the IT Switch in human 20S proteasome

While the IT switch is clearly important in archaea proteasome, does it play a role in regulating the H20S? To answer this question, we compared the sequence of the T20S to the 7 different H20S α subunits and determined if similar IT switch function was conserved in the H20S and H26S proteasome. We found that indeed there is a high conservation of the IT switch motif (e.g., a hydrophobic residue paired with a polar residue) in all α subunits except α3, which instead has a conserved pair of threonine residues (i.e., “TT” instead of “IT”; SFig 7B). In addition, this IT motif is separated from the critical YDR motif by exactly one residue in T20S and all seven human α subunits. Additionally, the critical Pro17 is 3 residues away from the IT switch in all cases. Interestingly, we found that α4’s N-terminus is far more conserved with the N-termini of the T20S α subunit then any of the other human α N-termini (SFig 7C&D), and correspondingly that α4’s IT switch is identically conserved with I and T residues. This is noteworthy because the T20S α N-termini all participate in gate closing and opening, since it is a homoheptamer, while primarily the N-termini of the heterohexameric α2, 3, and 4 subunits in the H20S participate in gating. Together this conservation of the IT switch in H20S, and particularly the strong conservation in the α 4 N-termini, which plays a significant role in the closed gate, suggests that it may play a key role in regulating gate-opening in the H20S.

To assess the potential functional role of the IT switch in H20S gating, we analyzed structural changes that occur upon gate-opening in the 26S proteasome when the 20S is closed (E_A1_) and open (E_D2_;)^18^ states (PDB: 6MSB & 6MSK). The N-termini of α’s 2, 3 and 4, are the primary ones that undergo opening and closing, although α1 and α5 do make small conformational changes and contribute to the closed state. We found that the IT switch functions in α2 (LT) and α4 (IT) the same way it does in the T20S—with the “T” under Helix 0 in the closed state and switching to the hydrophobic residue (I or L) under Helix 0 in the open state (SFig. 7F; summarized interactions in SFig. 7E). This indicates the IT switch is indeed functionally conserved in the H20S. Interestingly, the residues corresponding to the IT switch on α3 (TT) function a bit differently. The α3 T10 residue is out of the Helix 0 pocket in the closed and open states (SFig. 7F) but the α3 T9 side chain function similar to the I12 in the T20S and moves into the pocket under Helix 0 when the 20S is activated. When we mutated the homoheptameric T20S IT switch to resemble the IT switch of α3 (i.e., T20S-I12T to make a “TT” pair), this mutation appeared to destabilized I12 interaction with its neighbor in the closed state. However, the α3 T9’s neighbor in the closed state does not present a hydrophobic pocket, but rather another threonine (α2-T12), seemingly promoting a stable closed state with a T in this normally hydrophobic IT switch position. As for α6 and 7, they are always in the open state, and both of their hydrophobic IT switch residues stay in the position under Helix 0 as expected for IT switch function. Lastly, α1 and 5 both interact, to a small degree, with the α2, 3, 4 closed state and their N-termini do move to a more “open” state upon activation. Consistent with this, α5’s hydrophobic IT switch residue moves in under Helix 0 in the open state also consistent with IT switch function. However, α1’s IT switch is a clear outlier, it’s T14 IT switch residue (at least in these models) stays in place under Helix 0 in both states. In conclusion, the IT switch function uncovered in the T20S is well conserved in sequence and presumably function in the H20S for 6 of the 7 αsubunits, especially when focusing on the role of the hydrophobic residue switching to the Helix 0 pocket, as we found in the T20S.

### Mechanistic differences between HbYX-dependent (ZYA) and HbYX-independent (PA26) induced gate opening

**Figure 5:**
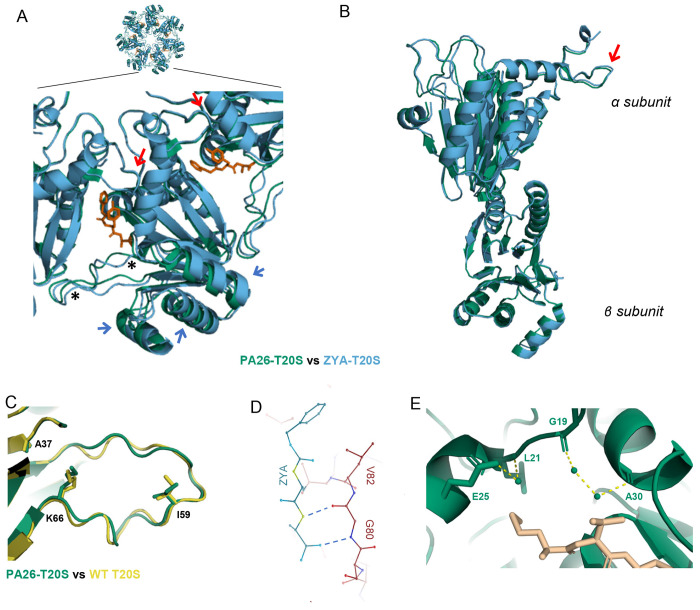
The HbYX motif (ZYA) induces gate-opening by different mechanistic principles than does PA26. **A.** Top view overlay of α subunits only from PA26-T20S (green) and T20S-ZYA (blue). Red arrows show Pro17 loop. Misalignments show that different conformational changes are present in ZYA bound versus PA26 bound states, e.g. alpha helixes (blue arrow), and differences in back loops (*). **B.** Overlay of PA26-T20S (green) and T20S-ZYA (blue) α and β subunits, aligned by β subunit. Red arrow shows Pro17 loop. **C.** View of loop proximal to K66, including overlay of PA26-T20S (green) with WT T20S (yellow) with key residues shown in sticks. **D.** ZYA forming a β-sheet-like interaction with residues, V82 and G80. **E.** Model of PA26-T20S showing interactions between PA26 C-terminus with waters (spheres) and T20S α residues (sticks).

To elucidate how HbYX (i.e., ZYA)-induced gate opening differed from PA26 induced gate-opening, we compared our ZYA-T20S model against the PA26-T20S model (PDB:1YA7) (Fig. 5A&B). Both activators show key commonalities and six significant differences in the way they appear to induce gate-opening. Both ZYA and the C-termini interact with the base of the intersubunit pockets via β-sheet like H-bonding (Fig 2M and 5D)^9^. More importantly, both ZYA and PA26 trigger conformational change in the IT switch to stabilize gate opening (Fig 4), and both cause the Pro17 to move away from the central pore “pulling” on the IT switch. The primary difference in mechanism appears to be: **1)** how the Pro17 gets moved away from the pore. When PA26 binds to T20S, its activation loop displaces Pro17, without inducing any rotation in the α subunits^9^. However, when ZYA binds, a different conformation is seen in the α ring. We overlayed the T20S α ring from the PA26-bound and ZYA-bound states to see differences in the “gate-open” states induced by these two activators (Fig 5A&B). Notice that Pro17 is, as expected, in similar positions, however the areas of non-overlap show differences in conformation of the 20S activated states. For example, relative to PA26 binding, ZYA-binding causes the α subunits to rotate around the radial axis (SMovie 1), which is clearly visible in Fig. 5A (blue arrows). Interestingly, this ZYA-induced α rotation (combine with intrasubunit conformational changes) causes the Pro17 displacement that appears to trigger the IT switch to the open state (Fig 2). **2)** ZYA-binding also rearranges K66 (Fig 2J versus Fig 5C), but PA26 binding does not appear to do this. This intriguing K66 rearrangement appears to be due to, **3)** the limited length of the intersubunit “bridging” of the YX residues in the HbYX motif described above (Fig 2M), combine with the backbone H-bond network stabilizing this position (Fig 2N). We imagine that this lack of intersubunit “bridging” across the pocket explains why PA26 does not cause the α ring rotation that we observed in HbYX-dependent gate-opening. **4)** The K66 adjacent back-loop gets rearranged by ZYA binding, but not by PA26-binding (Fig 5A, see asterisks), which is also clearly seen by comparing Fig 2J and Fig 5C (presumably due to K66 reorganization (Fig2N). **5)** ZYA-binding rearranges water molecules in the intersubunit pockets differently than does PA26-binding (Fig 2S versus Fig 5E). In fact, the lack of a tyrosine residue in the penultimate position meant that the C-terminus of PA26 did not displace or interact with the water molecules hydrogen bonding with the backbone of G19 and A30 (Fig. 5E). Additionally, the lack of tyrosine meant that there was no hydroxyl group present to interact with the water already hydrogen-bonding to the side chain of E25 and the backbone of L21 (Fig. 5E). Thus, the binding of the HbYX motif appears to rearrange waters in the intersubunit pocket differently than does PA26’s C-termini. These 5 features we observe in the ZYA-bound T20S are not seen in the PA26-bound structure, leading us to conclude that while both mechanisms converge on IT switch activation, they each trigger the IT switch in separate ways. Collectively, these structures clearly distinguish the HbYX-dependent mechanism of activation from the PA26 mechanism, and highlight the causal interactions involved in HbYX-specific gate-opening and the associated conformational changes.

### ZYA activates mammalian proteasomes

**Figure 6.**
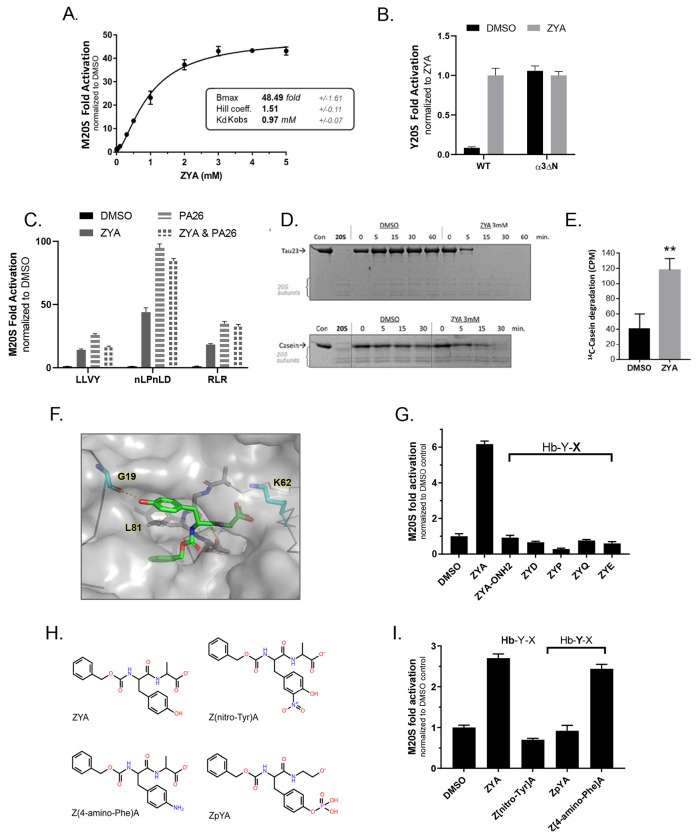
ZYA robustly stimulates gate-opening in yeast and mammalian proteasomes. **A.** Dose response of ZYA with mamalian 20S proteasomes (0.5 nM) and nLPnLD-AMC as substrate. Proteasome activity is normalized to DMSO control. Means were fit to the Hill equation, and equilibrium binding coefficients are shown.* **B.** WT and gateless (α3ΔN) yeast 20S proteasomes (0.5nM) incubated with or without ZYA (2.5mM) and nLPnLD-amc.* **C.** Mamalian 20S proteasomes (0.5nM) alone or with ZYA (2.5mM), or PA26 (55nM), or both (see graph key). Proteasome activity measured using three different fluorogenic substrates preferentially cleaved by different 20S protease sites (LLVY-amc, chymotrypsin-like, nLPnLD-amc, caspase-like; LRR-amc, trypsin-like).* **D.** Mammalian 20S proteasomes (100nM) incubated with tau23 (2uM; truncated tau protein) or β-casein (1 uM). At the indicated times, the reaction was quenched by addition of SDS loading buffer and separated by SDS-PAGE. Proteins visualized with Coomassie brilliant blue. Gels are representative of three independent experiments. 20S proteasome subunits are indicated with brackets to serve as loading controls for each sample. **E.** ^14^C casein was incubated with mammalian 20S proteasome similar to D for 30min at 37°C. Acid soluble counts after TCA precipitation were quantified via scintillation to show generation of peptide products.* **F.** ZYA (green sticks) docked in intersubunit pocket between α5&6 in human 20S. Image rendered in PyMOL. **G.** Mamalian 20S proteasome (0.5nM) activity (nLPnLD-amc hydrolysis, rfu/min) with the indicated ZYA derivatives (500uM) with variations in the “X” position that were found to be deleterious to ZYA activity. Proteasome activity is normalized to DMSO.* **H.** Structures of derivatives tested in I. **I.** Mamalian 20S proteasome activity (nLPnLD-amc hydrolysis, rfu/min) with the indicated ZYA derivatives at a low binding concentration of 100 uM. Proteasome activity is normalized to DMSO.* *Data (means) are representative of three or more independent experiments each performed in triplicate. Error bars represent ± standard deviation for panels A, B, C, E, G, and I.

We sought to determine if our findings with ZYA on the *Thermoplasma* 20S would translate to the mammalian system; therefore, we tested Ac-LYR, Ac-LYA, and ZYA on M20S. In agreement with the T20S outcomes, we observed significant improvements in gate-opening efficacy with each modification (SFig. 8A). ZYA exhibited the greatest effect on the M20S, and a dose response with ZYA showed nearly 50-fold activation at saturating levels (Fig. 6A vs SFig. 8B). Although ZYA can stimulate the M20S activity robustly, it has low affinity with a k_obs_ of ~1mM (Fig. 6A), which is highly similar to its affinity for the T20S (Fig. 1J). To confirm that ZYA activates via gate opening in eukaryotic proteasomes, we asked if ZYA could activate the yeast open-channel mutant (α3ΔN) 20S. We found that ZYA could not activate it at all but did activate WT yeast 20S as expected (Fig. 6B), consistent with analogous experiments in the archaeal system. Interestingly, the saturation curve for ZYA-induced proteasome activity for the M20S gave a cooperative binding curve with a significant hill coefficient of 1.5 +/−0.1 (Fig. 6A) This indicates that ZYA binding is cooperative and that binding to more than one site occurs during the allosteric induction of gate-opening. This is consistent with published cryo-EM structures of the 26S^18,19^ that showed multiple C-termini binding before the open-gate state is fully stabilized. While the minimal number of HbYX motifs required to induce gate opening is unknown, the hill coefficient of 1.5 suggests that a minimum binding of two molecules is involved.

We next tested the ability of ZYA to stimulate the M20S to hydrolyze three different peptide substrates that are preferentially cleaved by 20S’s three different protease sites (LLVY-amc, β5; nLPnLD-amc, β1; LRR-amc, β2). We observed a significant. 10- to 50-fold increase, in the hydrolysis rate of all three peptide substrates (Fig. 6C, compared to DMSO), which is expected for a gate opener. In addition, to determine how well ZYA was able to induce gate-opening, we compared it to M20S activation by PA26. Saturating PA26 concentrations typically stimulate M20S activity by 30-100-fold, depending on the basal activity of the 20S preparation. We found that ZYA was able to stimulate the M20S similar to that of PA26: ~50-fold activation by ZYA and ~90-fold for PA26 (for nLPnLD-AMC). In addition, when we combined ZYA and PA26 in a single reaction there was no synergy between these activators. Instead, there was a slight decrease in activity, relative to PA26 alone, which is likely due to expected competition between ZYA and PA26 binding to the intersubunit pockets. Collectively, these results demonstrate that ZYA is a highly effective and robust gate-opening activator of the M20S proteasome from archaea, yeast and mammals. These results support the hypothesis that this HbYX peptide mimetic functions analogously to the highly conserved HbYX motif.

### ZYA stimulates protein degradation by the mammalian 20S proteasome

We have demonstrated that ZYA robustly activates peptide hydrolysis via gate-opening but what about that 20S’s capacity to degrade unstructured proteins? To answer this question, we asked if ZYA could stimulate the M20S proteasome to degrade tau23 (a truncated tau protein that is found in brain) and the model unstructured protein—casein. ZYA significantly increased the degradation of both proteins, as visualized by SDS-PAGE with Coomassie stain (Fig. 6D). Tau and casein appeared to be completely degraded within the first 15 mins and 30 mins, respectively. In support of these results, we also measured the peptides generated from the degradation of ^14^C-casein by the 20S in solution (Fig. 6E) as measured by acid-soluble counts. In agreement with the gel-based protein degradation assay, ZYA significantly increased the number of soluble peptides from the degradation of ^14^C-casein. These results clearly demonstrate that ZYA can robustly stimulate the degradation of unfolded proteins.

### Probing structure activity relationships of the ZYA gate-opening compound

To elucidate how ZYA might be binding to the human proteasome, we computationally docked ZYA in the human α5/6 intersubunit pocket, where the HbYX motif of Rpt5 binds. Our docking results (Fig. 6F) suggest that ZYA binds in a similar configuration to the HbYX motif of various PAs (Fig. 2M), and as reflected in our ZYA-T20S structure. To evaluate the specificity and HbYX motif-like requirements of ZYA for the M20S, we proceeded to probe ZYA’s structure/function relationships and efficacy through chemical modifications (Fig. 6G&I). First, we asked if additional negative charges in the “X” position of the HbYX motif could be tolerated by replacing alanine with acidic residues. We found that ZYE and ZYD failed to activate the M20S (Fig. 6G). We also tested if causing a backbone torsion constraint (ZYP) and a polar group (ZYQ) might stabilize the peptide’s structure and binding, but they too abrogated M20S stimulation activity (Fig. 6G). Prior studies and sequence conservation showed that small, medium, and large aliphatic, and basic residues could all be tolerated in the “X” position, thus the “X—variable” designation^16^. Our data with the ZYA also suggests there are some limitations (i.e., negative charges) in this position. Prior studies^9,16,27^ also showed the HbYX motif’s terminal carboxy group forms an ionic bond with K66. To validate that this was important for ZYA function in M20S, we blocked its C-termini carboxyl with a NOH2 group (ZYA-[NOH2]). Carboxy blocking completely abrogated ZYA activity (Fig. 6G), as expected for canonical HbYX motif function. Our ZYA-T20S structure indicated that bridging of the intersubunit pocket by ZYA contributes to inducing conformational changes that cause gate opening. To test this hypothesis in the M20S, we modified the tyrosine of ZYA to lengthen the “bridging” distance by adding a nitro or phospho group to the tyrosine hydroxyl, which would lengthen the bridging distance by 1 or 2 bonds respectively (Fig. 6H). Both ZpYA and Z(nitro-Tyr]A failed to activate the 20S (Fig. 6I), supporting the bridging length requirement between the tyrosine hydroxyl and carboxy C-termini of ZYA. This conclusion is further supported by the fact that Z(4-amino-Phe)A still activates the 20S to a similar extent as ZYA (Fig. 6I), since it is similar in bridging length to ZYA.

### Comparing ZYA to known proteasome-activating small molecules

**Figure 7:**
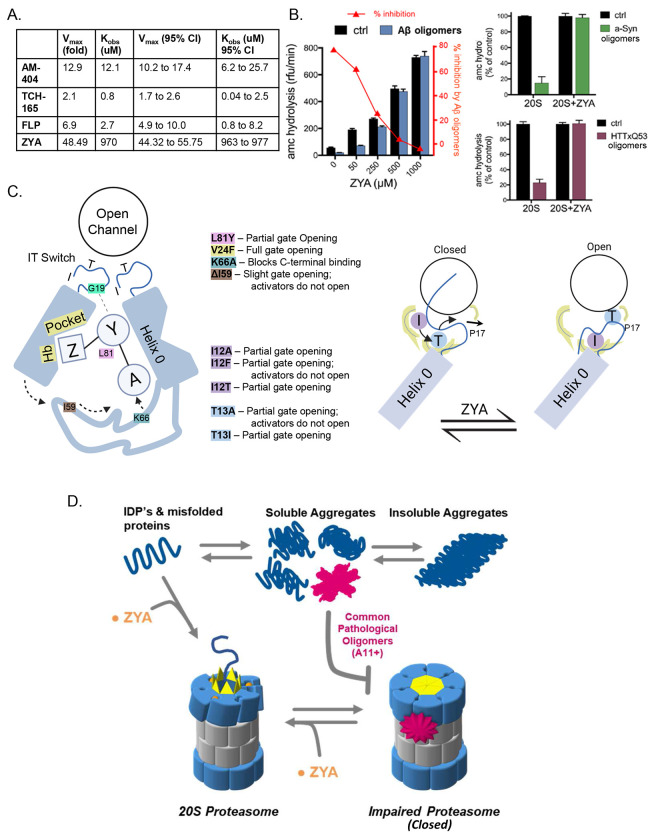
ZYA is a robust gate opening compound, that prevents inhibition by soluble oligomers, and provides a mechanistic framework to understand HbYX-dependent proteasome activation. A. Comparison of proteasome stimulation capacity and affinity of other known 20S stimulating compounds compared to ZYA. Values derived from saturation curves in Supplemental Figure 8B. Equilibrium coefficients were calculated from triplicates saturation curves and 95% CI are shown. B. (Left) 20S proteasome (0.5nM) activity (nLPnLD-amc hydrolysis, rfu/min) with ZYA at the indicated concentrations, with and without Aβ oligomers (0.5 uM) oligomers (Thibaudeau *et al.*, 2018). (Right) 20S proteasomes incubated with and without α-Syn (top) or HttQ53 (bottom) oligomers, and with or without ZYA (1mM). Rate of nLPnLD-amc hydrolysis is normalized to the control. Data (means) is representative of three or more independent experiments each performed in triplicate. Error bars represent ± standard deviation. C. (Left) Summary Figure of HbYX motif interactions with intersubunit pocket. Color key shows residues that were mutated in this study and summarizes their effects on ZYA function. (Right) Schematic demonstrating the function of the IT switch (I12, T13) that stabilizes the open and closed states of the proteasome gate. Functionally the IT switch is allosterically tethered to the Pro17 position, which is affected by the binding of ZYA and likely all proteasome activators that induce gate-opening. A color key shows residues that were mutated in this study on the IT switch and summarizes their effects on the function of the gating in the T20S proteasome. D. Small molecules that function similar to ZYA, have the potential to function at two different beneficial stages of protein degradation: 1) to upregulate the degradation of unstructured proteins that could misfold, oligomerize, aggregate, and become toxic, and 2) to restore normal proteasome function to proteasomes that could be impaired by toxic oligomers.

Recently, several studies have been published on the potential of small molecules to activate the 20S proteasome though their mechanisms of activation are still not clear. We compared how ZYA, designed to mimic the HbYX motif, performs relative to some of these small molecules (AM-404, TCH-165, Fluspirilene (FLP) ^30,31^). The other small molecules exhibited higher binding affinity relative to ZYA (with K_obs_ values in the micromolar range; Fig7 A). However, as indicated by V_max_ values, none of them activated the human 20S to the extent that ZYA was capable of even at saturating concentrations (Fig. 7A; SFig. 8B). Interestingly, none of the other compounds significantly activated the T20S or any of its variants (e.g., WT, αΔN, K66A) (SFig. 8C). Notably, ZYA activates WT T20S, but not the T20S-αΔN or T20S-K66A, as expected for a HbYX mimicking compound. This indicates that ZYA functions via the conserved HbYX-dependent mechanism, while the other compounds tested here do not appear to function by this conserved mechanism, since they do not affect the archaeal 20S. The ability of a small molecule such as ZYA to activate the proteasome nearly 50-fold with similar efficacy as the multimeric PA26 11S activator is, to our knowledge, unprecedented.

### ZYA abrogates the proteasome-inhibiting activity of three different A11+ oligomers

We recently published a study demonstrating that soluble oligomers of Aβ, α-synuclein, and huntingtin(Q53), which share a common tertiary conformation (A11+), allosterically inhibit 20S function by stabilizing the closed gate conformation^20^. Since ZYA stimulates gate opening, we investigated the possibility that it could also rescue the M20S from inhibition by these neurodegenerative-associated oligomers. Remarkably, when M20S activity is measured in the presence of oligomers, the addition of 1mM ZYA completely blocks inhibition by A11+ oligomers (Fig. 7B). Furthermore, even at 50μM, ZYA stimulates oligomer inhibited M20S enough to bring its basal activity back to WT levels (compare control with no ZYA to Aβ oligomers at 50μM ZYA). This is an unequivocal demonstration for the potential of small molecules to restore proteasome activity in conditions of M20S impairment. In addition, compounds that function like ZYA could also enhance degradation of intrinsically disordered proteins (IDPs) (Fig. 6D&E), which are often found to be the aggregation prone proteins involved in neurodegenerative diseases.

## DISCUSSION

Proteinopathies are associated with many diverse human diseases (e.g., Alzheimer’s disease, cardiomyopathies, and type II diabetes) and characterized by the accumulation of intracellular (and sometimes extracellular) misfolded proteins. Many of these misfolded proteinopathy-associated proteins are IDPs^31–33^, efficiently degraded by the 20S proteasome *in vitro*. Why the cells’ protein degradation systems are insufficient to degrade the intracellular proteins in NDs is not clear, although many NDs have been reported to exhibit abnormally low proteasomal activity, despite relatively normal amounts of cellular proteasomes^2^. In fact, for decades now, research has implicated impairment of the proteasome system in the etiology of NDs such as Alzheimer’s and Parkinson’s^3–7^ though the specific causes remain elusive. The function of the UPS is critical for the healthy maintenance of the proteome in general, and more specifically, for neuronal synapse function (i.e., plasticity and synaptic protein turnover)^1,35^. Based on our prior findings of oligomer impairment^20^, we generated the first multicellular organism (*C. elegans*) with a constitutively open 20S proteasome, that could not be inhibited by these toxic oligomers^36^. These worms with “hyperactive” 20S proteasomes have increased longevity and resistance to various proteotoxicities, including heat shock and oxidative damage. Moreover, increasing proteasome amount or activity in mammalian cell culture and multicellular organisms by various means has also proven feasible, and to increase degradation of endogenous and ND related proteins^36–41^. Together, these results motivated the current study to further elucidate the mechanism of how HbYX-dependent proteasome activators induce 20S gate-opening and determine the pharmacological tractability of this mechanism to potentially treat the aforementioned pathological proteinopathies.

Several cryo-EM structures of the 26S^18,19,23,24^ have been generated in the closed and open-gate states but these structures alone could not explain how the HbYX motif induces gate-opening. This is in part due to the complexity and dynamics of the interaction interface (described in the introduction) that encompasses 7 different 20S α-subunits and six different 19S ATPases subunits (RPT 1-6). To circumvent this mechanistic complexity, we first focused on the T20S from archaea, which is homoheptameric (i.e. mechanistically simpler), and contains the same conserved structural gating elements (e.g. N-terminal YDR motif, Pro17-reverse turn, closed and open states). Based on the structure of various HbYX motif’s bound to the proteasome (Fig 1), the results of various T20S mutations, and a systematically developed peptides, we sought to develop a minimal component of the HbYX motif—the peptide mimetic Z-YA. ZYA could only activate T20S with a functioning gate, validating it as a bona fide gate-opening compound (Fig 1). The 1.9Å cryo-EM structure of ZYA bound to the T20S also showed that it bound to the intersubunit pockets precisely the same way as the HbYX motif, establishing ZYA as a minimal HbYX mimicking compound. We further probed all three sub-binding pockets of ZYA to determine the potential mechanistic contributions of the Hb, Y, and X positions, in inducing gate opening. Combining the results from the mutagenesis and structures allowed us to develop a mechanistic model for how the HbYX motif (or ZYA) is able to induce gate opening and uncover a new important component of gating function—the IT switch, whose function is summarized in Figure 7C.

By comparing the structures of WT T20S (with newly resolved N-terminal residues), with ZYA-T20S, T20S-αL81Y, and previously published PA26-T20S, we develop a detailed molecular model of how the HbYX motif induce gate opening without contacting Pro17 (as PA26 does with its activation loops^9^). In this model, the HbYX Tyrosine-Alanine connects two neighboring α subunits by its tyrosine H-bonding to G19 and alanine carboxy salt bridging with K66 in the neighboring α subunit. While this interaction has been noted previously, at this higher resolution we observe a shift in the K66 side chain (Fig. 2J), it’s stabilization by a network of ionic interactions (Fig.2N), and a rearrangement of this loop at the back of the intersubunit pockets (Fig. 2J&N; D57-I67 or “back loop”) which is naturally more mobile (SFig. 2 and 4). The length of this “bridge” appears to be critical for gate-opening (Fig 6I), which supports the model that this back-loop reconfiguration is important for gate opening. Moreover, K66 and a back-loop of proper length (Fig. 2K) is required for gate-opening (Fig 1F), as the tyrosine by itself, T20S-αL81Y, did not induce full gate-opening or α subunit rotations. Combined, these indicate an important role of the “bridge” and the “back loop” for inducing α subunit rotations that cause gate-opening in the ZYA-bound state.

It is our working model that the reorganization of the back loop, which allows for the neighboring Helix 0 to rotate towards its neighbor, sets off an allosteric chain reaction around the α ring. When Helix 0 rotates away from its’ neighbor’s N-termini, it causes the neighboring Pro17 to also move towards this helix to maintain its packing interaction, leading to both inter and intra subunit induced conformational changes in the Pro17 position (Fig. 2I, L and Fig 7C). The repositioning of Pro17 then displaces the IT switch T13, pulling it out of the IT switch binding pocket under Helix 0. This T13 displacement then leaves the binding pocket empty allowing the more hydrophobic I12 to now bind under Helix 0, which reconfigures the gate and positions the N-termini into a compatible position for the YDR motif to stabilize the open state. In fact, this 1.9Å ZYA-T20S structure now shows the open state resolved up to G4.

An additional layer to this “back loop” rearrangement model should also include the importance of the bulky benzene group (Z) in ZYA. Mutations that placed a bulky aromatic in the Hb binding pocket (i.e. V24F, A154F, and V24Y; Fig 2O, P, Q, and R) all resulted in substantial gate-opening, especially V24F. These mutants suggest that HbYX motifs with a bulky Hb position may be strong activators of T20S gate-opening. However, a high-resolution structure of one of these mutants will be needed to determine the mechanism of gate-opening by aromatic ring occupancy in the Hb binding pocket. V24F could induce gate-opening by changing the Helix 0 position, as we saw with the αL81Y mutation, or it could somehow induce α subunit rotations like ZYA does. Further study is needed to confirm contribution to gating by the Hb pocket, though comparing activation by LYA versus ZYA provides further insight. Both small peptides could induce gate opening in the T20S (Fig 1J), but LYA presents a non-bulky aliphatic side chain to the Hb binding pocket, while ZYA presents a bulky aromatic. The fact that LYA could induce gate-opening similar to ZYA (though at lower affinity) indicates that perhaps intersubunit bridging by the YA is sufficient to induce gate opening. However, providing a bulkier Hb group increases affinity and likely efficacy, which was also a topic in a recent structural study from the Gestwicki group that used a chimeric PA26-HbYX complex^28^. Taken all together, the Hb group likely plays an important role in increasing affinity for the intersubunit pocket but likely also plays a direct mechanistic role in helping induce gate-opening. Therefore, the effects of the Y-A bridge that reorganize the back-loop and the interaction of Hb group under Helix 0 (especially if a bulky aromatic) combine into a minimal motif that can robustly induce proteasome gate opening in 20S proteasomes from archaea, yeast, and mammals.

Further structural studies will need to be done to determine how ZYA-induced gate opening occurs in the mammalian 20S, which has 7 different intersubunit pockets. However, our analysis of the 3-4Å H26S cryoEM structures in the open and closed states shows strong sequence and functional conservation of the IT switch mechanism (mapped out in SFig 7). Since the HbYX is conserved and ZYA functions similarly in T20S, Y20S, and M20S, we expect that similar mechanisms will be at play, though in a more asymmetric manner since primarily only α2, α3 and α4 contribute to the closed state, while the others are open. The H19S has been observed to engage with the H20S using all its three HbYX motifs on Rpt-2, −3, and −5; yet in the closed 26S state gate opening does not occur. Building on our current model, we speculate that this may be because HbYX subunits Rpt3, and 5 interact with α subunits that have their N-terminal tails already in a constitutively open conformation (α1 and α5). What about Rpt2, which interacts with α3? α3 has a modified IT switch (TT instead of IT) and the I12T mutation in T20S (TT) was more open than WT but couldn’t be activated by PA26 or PAN. This is consistent with α3 whose IT switch is not engaged under Helix 0 stabilizing its closed state; instead, α3’s N-termini is stabilized by sitting on top of and interacting with the closed N-termini of α2 and α4 (SFig 7B)^18^. Based on this unique α3 IT switch, and N-terminal position in the closed state, it’s perhaps not surprising that α3 appears to be desensitized to HbYX binding. Conversely, Rpt1 binding to α4 in the activated H26S state (SFig 7) has been linked with gate opening^18^, and it carries a partial HbYX motif (-TYN). Interestingly, α4 has perfectly conserved IT switch, and its N-terminal 1-34 residues is uncannily highly conserved relative to the T20S α subunit, more so than any other H20S α subunit’s N-termini (SFig. 8B). These two conserved elements of α4s gating region suggest a unique mechanistic importance for α4 and Rpt1 in controlling gate-opening in the eukaryotic 20S. Perhaps Rpt1s effect on the α4 subunit alone is sufficient to trigger α4s IT switch, also affecting α3 and 2, leading to gate opening, or perhaps contributions from other HbYX motifs are needed to trigger an allosteric system. Further study is needed to test this hypothesis based on the findings presented here. Nevertheless, it is apparent that the eukaryotic 26S gating system evolved a spectrum of Rpt C-termini sequences, IT switches, and α N-termini to fine tune how gate opening is controlled by substrate binding to the 26S proteasome. We expect that the identification and function of the IT switch and the other HbYX relevant mechanism defined here for the T20S will guide new understanding of how these mechanisms regulate the more complicated 26S proteasome as suggested here.

Interestingly the mechanism uncovered here also shed light on how oligomers could impair the 20S proteasome, without being able to enter the internal chamber of the proteasome^20^. If the HbYX mechanism requires a functioning back loop for activation, which is supported by our structure and the activity of the T20S-I59Δ, then this back loop could be a target for toxic oligomers that impair proteasome function by blocking HbYX dependent gate-opening but not PA26 induced gate-opening^20^. Our results here show that even at concentrations much less than saturating, ZYA is effective at reversing proteasome inhibition by three different ND-related proteins (Fig. 7B). We expect compounds that could similarly induce gate opening at more physiologically relevant affinities could potentially treat ND (Fig. 7D). Such compounds could both stimulate degradation of most ND-related proteins, which are typically IDPs, and simultaneously reverse proteasome impairment, which has been observed in aging. Advancements in understanding the proteasomes gate-regulatory mechanisms provide a framework for the development of small molecules that antagonize proteasome impairment, especially by oligomers, or activate its ability to degrade unstructured proteins. Considering the importance of the proteasomal gate in regulating protein degradation, gate-activating compounds (such as ZYA) have promising potential as research tools to probe proteasome function *in vitro* and possible *in vivo* to perhaps, elucidate the role of proteasome impairment in neurodegenerative disease progression.

## METHODS

### Proteasome purifications

*T.acidophilum* wild type (WT) 20S, Δα_2-12_, and all other mutant 20S proteasomes were similarly purified as described^42^, except via 8XHis tags on the C-terminus of β subunit. All 20S mutants were generated by overlapping PCR site-directed mutagenesis. The plasmid for *M.jannaschii* PAN(M74A), kindly provided by Dr. Peter Zwickl^30^, lacked a 6His-tag, and was purified as described^38^, but Tris buffers were made at 50 mM instead of 20mM. Mammalian 20S proteasomes were isolated from bovine liver as described^39^. WT and mutant α3ΔN yeast 20S proteasomes were expressed and purified by anion-exchange chromatography as described^40^. Human proteasomes, tagged with HTBH on β4 subunits, were affinity purified from HEK293T cells as described^43^ with slight modifications. Cells were resuspended in Lysis Buffer (50mM Tris, protease inhibitors) and sonicated. Lysates were centrifuged at 20,000 x *g* and supernatants were incubated with streptavidin agarose resin (Millipore) for 1 h at RT. The beads were then incubated with biotin tagged-TEV protease in 50mM Tris overnight at RT.

### Proteasome activity assays – peptide substrates

Fluorogenic substrate peptides were purchased from BostonBiochem (suc-LLVY-amc) and EZBiolabs (ac-nLPnLD-amc, ac-RLR-amc, LFP (Mca-AKVYPYPME-Dpa(Dnp)-amide)), PAN CT peptides, ZYA, and ZYA derivatives were synthesized by ABclonal. Compounds (AM-404, TCH-165, Fluspirilene (FLP)) were purchased from Cayman Chemical. Peptides and chemical compounds were dissolved in DMSO and incubated with proteasomes at indicated concentrations. The final concentration of DMSO in activity assays was 2%. Oligomers of Aβ*56, α-synuclein, and huntingtin-Q53 were prepared as described^20^. All oligomers used were recognized by the α-oligomer antibody, A11^42^. Protein concentrations were determined by Bradford assay (Thermo Scientific). To measure peptide hydrolysis, fluorogenic peptides dissolved in DMSO were used at a final concentration of 25 - 100 μM for Suc-LLVY-amc and Ac-nLPnLD-amc, and 3 -10 μM for LFP and Boc-RLR-amc, in 50 mM Tris (pH 7.5), 1 mM DTT. For archaeal 20S experiments the indicated concentrations of T20S and LFP peptide was added to the buffer at 45°C, and where not indicated, 1 μg of PAN and 10 μM ATPγS (+5 mM MgCl_2_), or 2mM ATP and 10mM MgCl_2_, was added to the 0.1 ml of reaction buffer (sufficient to saturate the 20S particles)^43^. Amount of archaeal 20S from different purification preparations is normalized to their rate of LLVY-AMC hydrolysis, whose degradation is not regulated by gating. Assays with mammalian (0.5 nM and 1 nM as indicated), yeast wild-type (2 nM), and yeast α3ΔN (0.2 nM) proteasomes were performed at 37°C. Assays with Aβ, α-synuclein, and huntingtin-Q53 oligomers were performed as described^20^. Assays were performed for 30mins to an hour and analyzed using BioTek Gen5 Data Analysis software.

### Proteasome activity assays – protein substrates

Tau23 (gift from Eckhard Mandelkow) or β-casein (Sigma) were incubated with mammalian 20S proteasomes for the indicated time at 37°C. Reactions were quenched by the addition of LDS sample buffer (Invitrogen). Proteins were separated by SDS-PAGE using NuPAGE^™^ 4–12% Bis-Tris protein gels (Invitrogen) and visualized with Coomassie brilliant blue. Degradation of ^14^C-Casein was assessed by scintillation of acid soluble counts as described in^44^.

### Cryo-EM Sample Preparation and Data Collection

Copper Quantifoil R 1.2/1.3 300 mesh (EMS) grids were cleaned using a PELCO easiGlow Glow Discharge cleaning system. A volume of 3 uL of 0.5mg/mL WT T20S, T20S-αL81Y or T20S with 4mM ZYA (suspended in 50mM Tris pH 7.4, 150mM NaCl) sample was placed onto a grid, and then flash frozen in liquid ethane using a manual plunge freeze apparatus. Data collection was done using a Titan Krios transmission electron microscope (Thermo Fisher) operated at 300kW and a magnification of x81,000, which resulted in 0.503Å/px. Images were collected using a Falcon IIIEC direct electron detector camera equipped with a K3/GIF operating in counting and super resolution modes. Electron dose per pixel of 50 e-/Å2 was saved as 40 frame movies within a target defocus range of −2.5 to −1.25. All the data was collected using cryoSPARC software (Structura Biotechnology Inc.)^45^.

### Cryo-EM Single Particle Analysis

Cryo-EM images of the WT, T20S-αL81Y, and ZYA-T20S proteasome were analyzed using cryoSPARC. Schematic for cryo-EM single-particle data processing available in supplement.

WT T20S: From 1744 movies collected, we picked 444,678 particles after four rounds of 2D classification, which were used to generate an Ab-initio model and processed through heterogenous refinement then homogenous refinement (using D7 symmetry).

T20S-αL81Y: From the 2850 movies collected, we used 2847 in analysis and picked 889,069 particles after three rounds of 2D classification to obtain the best particle sets. The particles chosen from 2D classification were used to generate an Ab-initio model, which was used for homogeneous refinement (using D7 symmetry).

ZYA-T20S: From movies collect, we used 830,572 particles after two rounds of 2D classification. Particles isolated were used to generate an Ab-initio model, processed in a heterogenous refinement, then homogenous refinement (using D7 symmetry).

Final map was imported into Phenix^46^ to run density modification (DenMod) from two half maps. All representations (figures and movies) of the T20S proteasome complex were created using PyMol 2.5.2, WinCoot 0.9.6 EL, and UCSF ChimeraX v1.3^47,48^.

### Atomic model building

The atomic models were built using a modified version of the T20S from PDB: 1YA7 as a template, rigid body fitting into the electron density map using PHENIX 1.19.2-4158. The docked models were subjected to a cycle of morphing and simulated annealing, five real-space refinement macrocycles with atomic displacement parameters, secondary structure restraints, and local grid searched in PHENIX. Consequently, the models were refined by oscillating between manual real-space refinement in WinCoot 0.9.6 EL and real-space refinement in PHENIX (five macrocycles, without morphing and simulated annealing). ZYA was docked using LigandFit on PHENIX. Waters were added to models using PHENIX Douse.

### Computational Docking

Automated docking of the ZYA peptide mimetic in human proteasome intersubunit pocket was done with Glide from the Schrodinger Suite^49^.

### Statistical analysis

Data were analyzed in Graph Pad or excel using an unpaired Student’s t-test (Prism). For all statistical analyses, a value of *p* < 0.05 was considered significant.

### Data availability

The authors declare that data supporting the findings of this study are available within the paper and its supplementary information files and are available from the corresponding author upon request.

## Supplementary Material

1
