# Supplementary material for: Minimal mechanistic component of HbYX-dependent proteasome activation
Source: Res Sq. 2023 Mar 20:rs.3.rs-2496767. Preprint. [Version 1] doi: 10.21203/rs.3.rs-2496767/v1 (PMC10055539; doi:10.21203/rs.3.rs-2496767/v1)
Supplement: 1 [file NIHPPrs2496767v1-supplement-1.pdf]

Supplemental Figures and Legends

Supplementary Figure 1: T20S-ZYA Processing Scheme

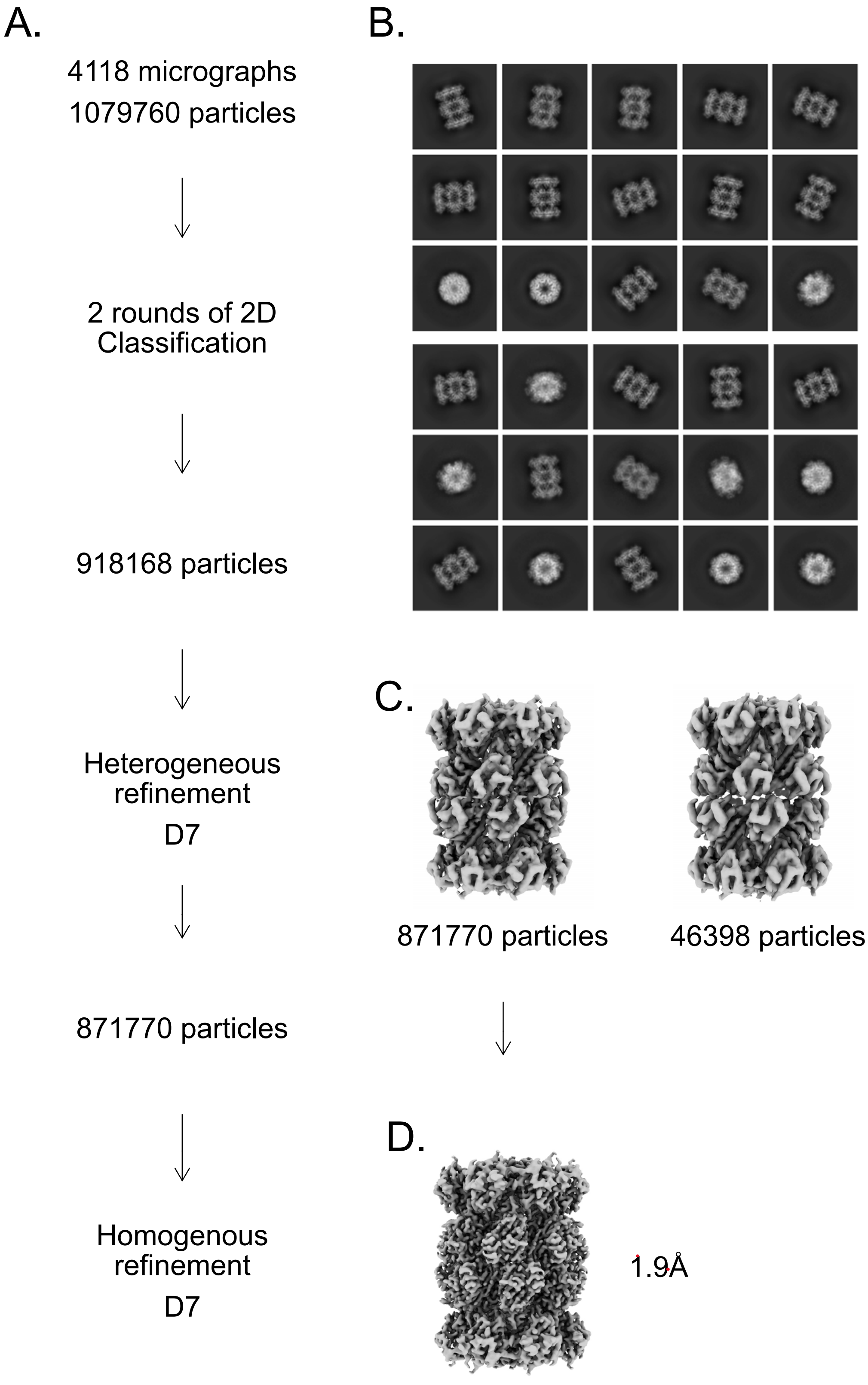

SFig. 1: T20S-ZYA Processing Scheme

A. Cryo-EM workflow for T20S-ZYA. All steps were performed in Cryosparc.

B. Final 2D class averages after 2 rounds of 2D Classification shown with a mixture of top and size views.

C. Maps of heterogenous refinement, performed to further separate T20S particles of varying conformations.

D. Homogenous refinement of class of particles that showed the open gate conformation with ZYA bound.

Supplementary Figure 2: T20S-ZYA Validation

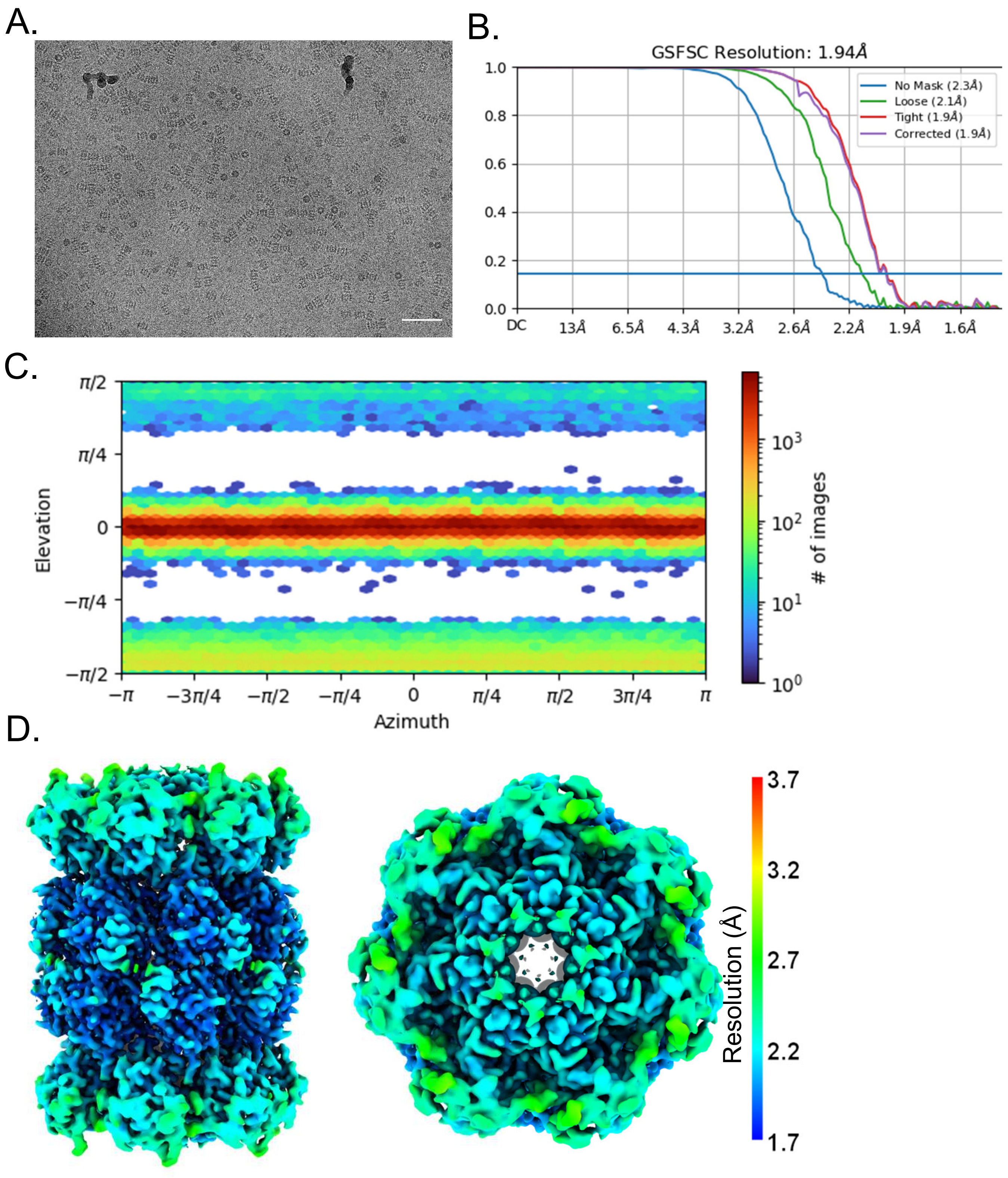

SFig 2: T20S-ZYA Validation

A. Micrograph representative showing T20S. Scale bar represents 5nm.

B. Standard FSC-0.143 graph showing correction 1.9A resolution.

C. View of particle angle distribution showing a mixture of side and top view particles.

D. Particles from selected ab-initio model were refined with homogenous refinement and D7 symmetry applied. D7 symmetry was confirmed as appropriate by homogenous refinement with C1 symmetry; the resulting C1 map was copied then flipped 180deg around the 2-fold axis then rotated 180 deg about the 7 fold axis. The flipped and rotated map was essentially identical to the original confirming D7 symmetry of the complex. Final 1.9A D7 map was colored in Chimera to show range of resolution of 3D reconstructed map. The chosen resolution scale is consistent for all three maps shown here to allow better comparison.

Supplementary Figure 3: WT T20S Processing Scheme

A.

1744 micrographs
940560 particles

↓

4 rounds of 2D Classification

↓

490458 particles

↓

Heterogenous refinement
D7

↓

444678 particles

↓

Homogenous refinement
D7

B.

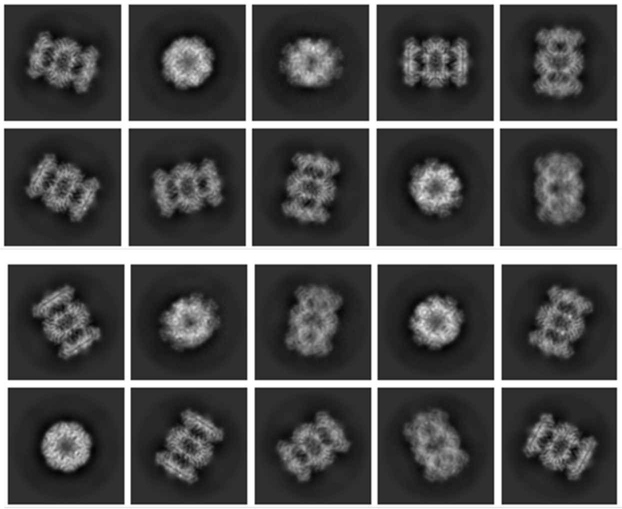

C.

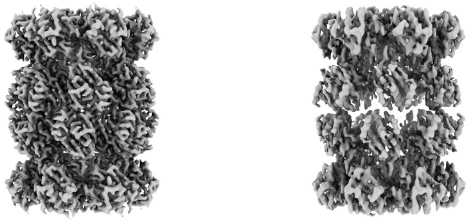

444678 particles

45780 particles

↓

D.

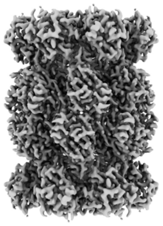

2.1Å

SFig 3: WT T20S Processing Scheme

A. Cryo-EM workflow for WT T20S.

B. Final 2D class averages after 4 rounds of 2D Classification shown with a mixture of top and size views.

C. Maps of heterogenous refinement, performed to further separate T20S particles of varying conformations.

D. Homogenous refinement of class of particles that showed the closed gate conformation.

Supplementary Figure 4: WT T20S Validation

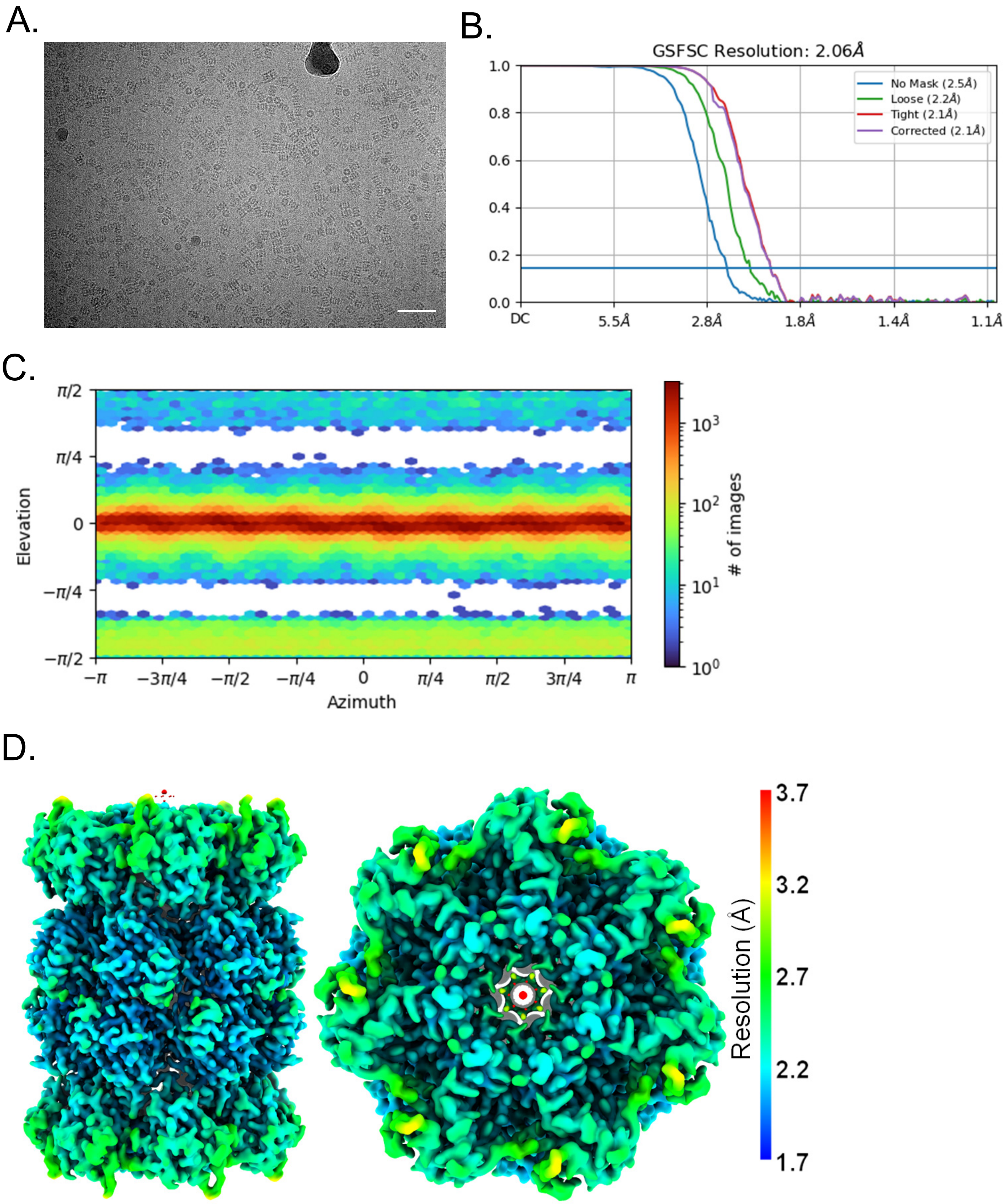

SFig 4: WT T20S Validation

A. Micrograph representative showing T20S. Scale bar represents 5nm.

B. Standard FSC-0.143 graph showing correction 2.06A resolution.

C. View of particle angle distribution showing a mixture of side and top view particles.

D. Particles from selected ab-initio model were refined with homogenous refinement and D7 symmetry applied (D7 symmetry application was confirmed as appropriate as discussed in SFig 2D). Final 2.12A map colored in Chimera to show range of resolution of 3D reconstructed map. The chosen resolution scale is consistent for all three maps shown here to allow better comparison.

Supplementary Figure 5: L81Y T20S Processing Scheme

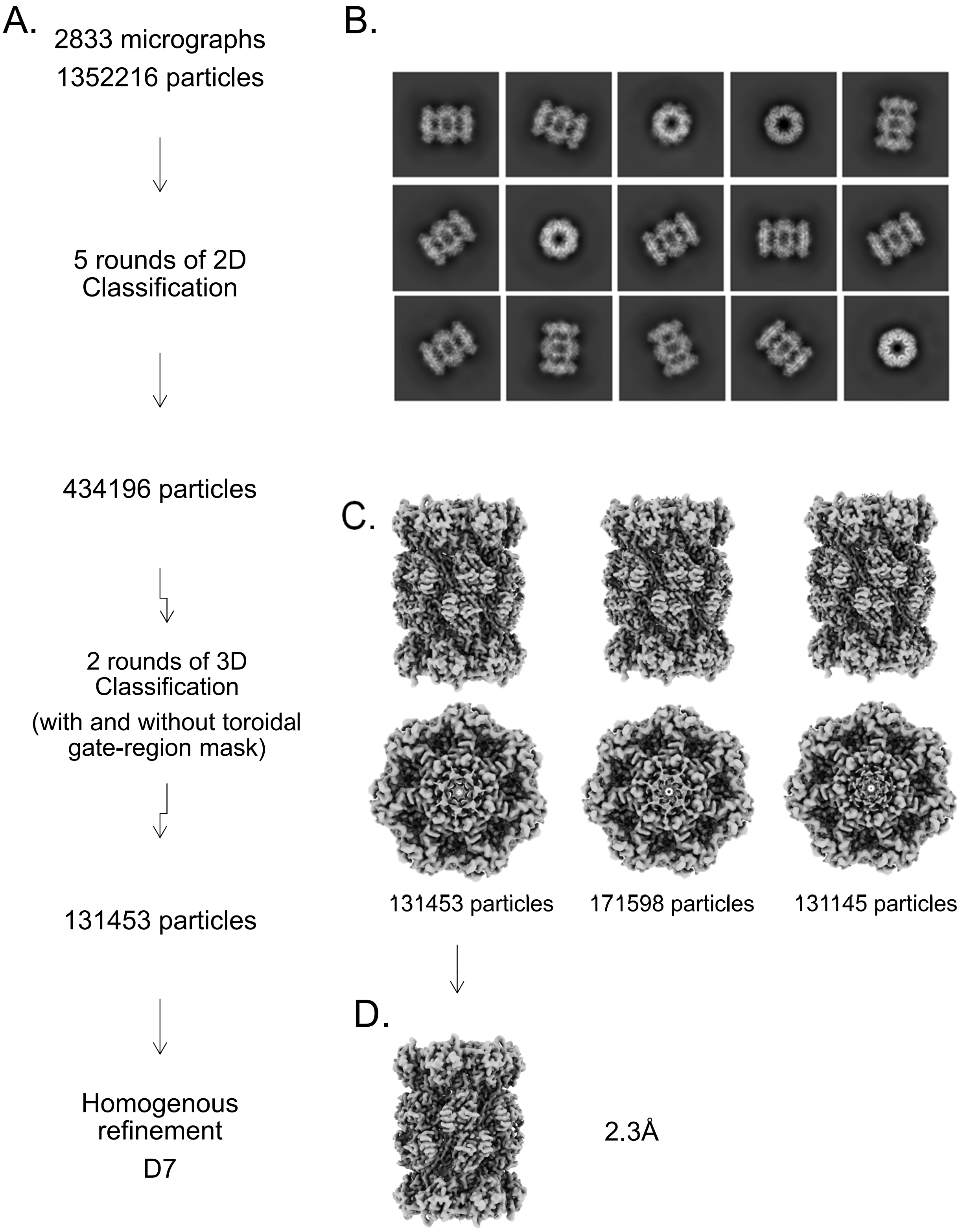

SFig 5: L81Y T20S Processing Scheme

A. Cryo-EM workflow for L81Y T20S.

B. Final 2D class averages after 5 rounds of 2D Classification shown with a mixture of top and size views.

C. Maps of 3D Classification, performed to further separate T20S particles of varying conformations at the gating residues.

D. Homogenous refinement of class of particles that showed the open gate conformation.

Supplementary Figure 6: L81Y T20S Validation

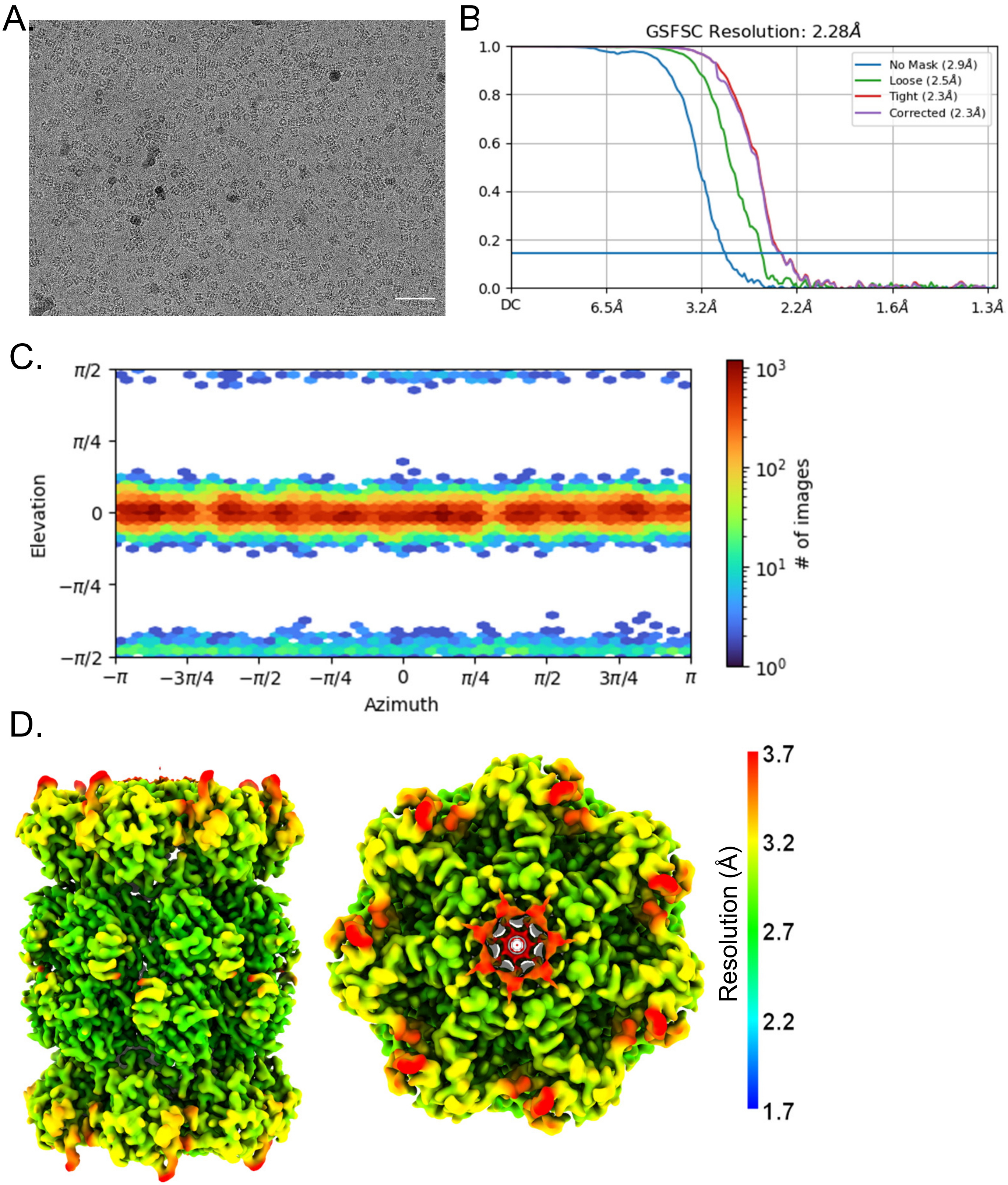

SFig 6: L81Y T20S Validation

A. Micrograph representative showing T20S. Scale bar represents 5nm.

B. Standard FSC-0.143 graph showing correction 2.28A resolution.

C. View of particle angle distribution showing a mixture of side and top view particles, with a higher proportion being side view.

D. Particles from selected ab-initio model were refined with homogenous refinement and D7 symmetry applied (D7 symmetry application was confirmed as appropriate as discussed in SFig 2D). Final 2.28A map colored in Chimera to show range of resolution of 3D reconstructed map. The chosen resolution scale is consistent for all three maps shown here to allow better comparison.

Supplementary Figure 7: SFig 7: IT switch is conserved from archaea to humans and its conformational state is tightly coupled to N-termini state (closed versus open) in the human 26S proteasome.

A.

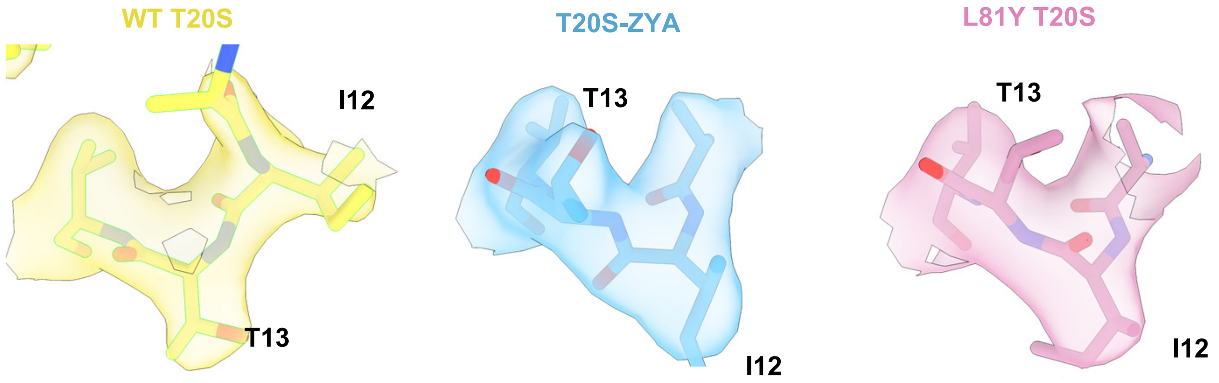

B.

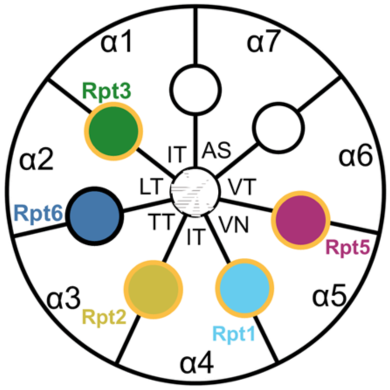

20S Core Particle		19S Regulatory Particle		PA200
α	N-terminal sequence	Rpt	Rpt C-term Sequence	C-term Sequence
1	…FDRH**IT**IFSP…	3	QEHE**FYK**	
2	…YSFS**LT**TFSP…	6	SIKKLWK	
3	…YDSR**TT**IFSP…	2	TPEG**LYL**	
4	…YDRA**IT**VFSP…	1	PRYMT**YN**	
5	…YDRG**VN**TFSP…	5	ANLQ**YYA**	VSPC**YYA**
6	…YDND**VT**VWSP…			
7	…YDLS**AS**TFSP…			

C.

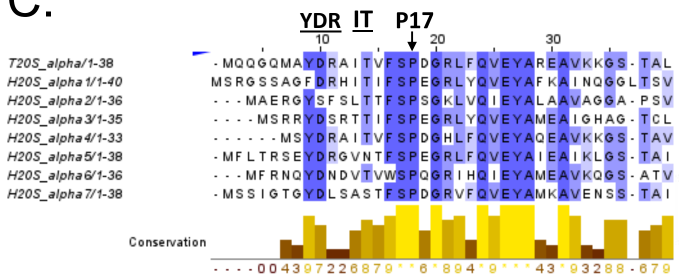

D.

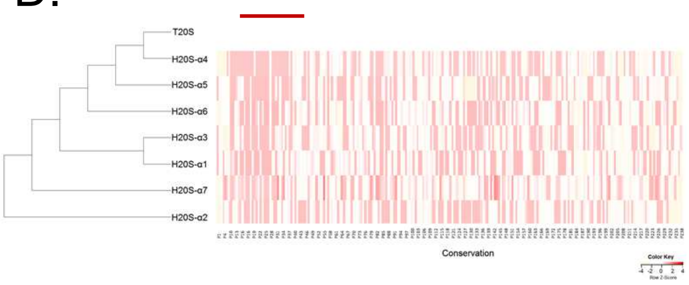

E.

Subunit	Residue under Helix 0 in **closed** state	Residue under Helix 0 in **open** state	N-termini switches states from closed to open
T20S α	T13	I12	Yes
α1	T14	T14	Partially
α2	T11	L10	Yes
α3	none	T9	Yes
α4	T8	I7	Yes
α5	none	V12	Partially
α6	V10	V10	Stays open
α7	A11	A11	Stays open

F.

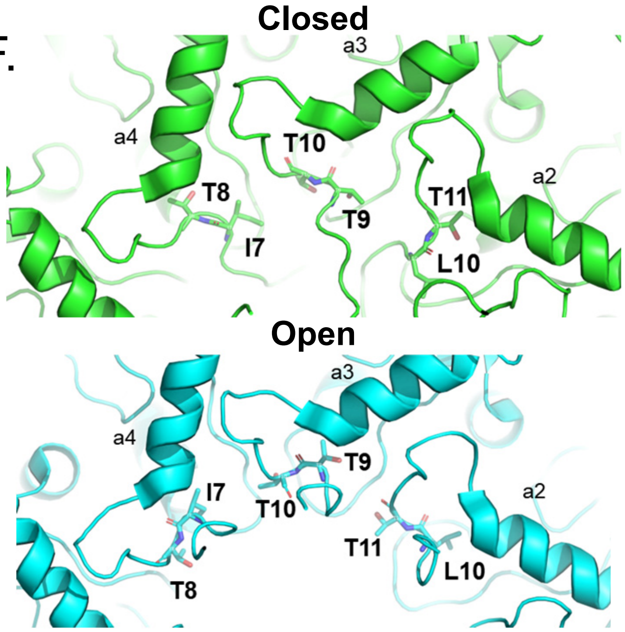

SFig 7: IT switch is conserved from archaea to humans and its conformational state is tightly coupled to N-termini state (closed versus open) in the human 26S proteasome.

A. Map and model (stick) of each indicated species of T20S, showing specifically the IT switch and how it is modeled to fit its corresponding densities.

B. (Left) Schematic of H20S engaged with H19S in the ED2 state (activated and gate-open) showing the corresponding IT switch residues around the pore and intersubunit pockets that engage with HbYX Rpt C-terminal tails. Grey hatched triangles in in pore schematize the N-termini from the corresponding alpha-subunit that contribute to forming the closed gate. (Right) N-terminal sequences of alpha subunits containing the IT switch (and surrounding) residues and the sequences corresponding to the 19S ATPases C-terminal tail, if engaged.

C. Alignment showing the conversation of sequences in the first 40 positions of the α subunits of the T20S and the H20S. Alignment was performed using ClustalΩ and conservation and figure was generated using Jalview 2.11.2.5. Purple highlights residues most conserved in a position and the bar graph in yellow indicates the conservation score, accounting for size, charge, hydrophobicity, and polarity.

D. Heatmap represents the conservation of the sequence compared to the T20S. For visualization, the conservation values of each amino acids were scaled to enhance and discern positions with similar values between subunits. The scaling was performed with Z score in R 3.6.3. Red represents highly conserved AA and the yellow to white represents low conservation values for that AA position. The tree was generated in MEGA with the UPGMA method which is constructed based on pairwise comparisons of the subunit's alignments. (This method assumes equal or highly similar mutation rates). Red bar represents region of high conservation between T20S and human 20S alpha-4.

E. Summary table of α subunits in T20S and H20S and their corresponding IT switch conformational changes. Compare switching states to N-termini involved in gating in B.

F. Comparison of α2, 3, and 4 IT switch residues in the closed and open states of 26S proteasome (PDB: 6MSB, 6MSK)

Supplemental Figure 8: ZYA's capacity to induce gate opening in human and archaeal 20S compared to other known small molecule activators.

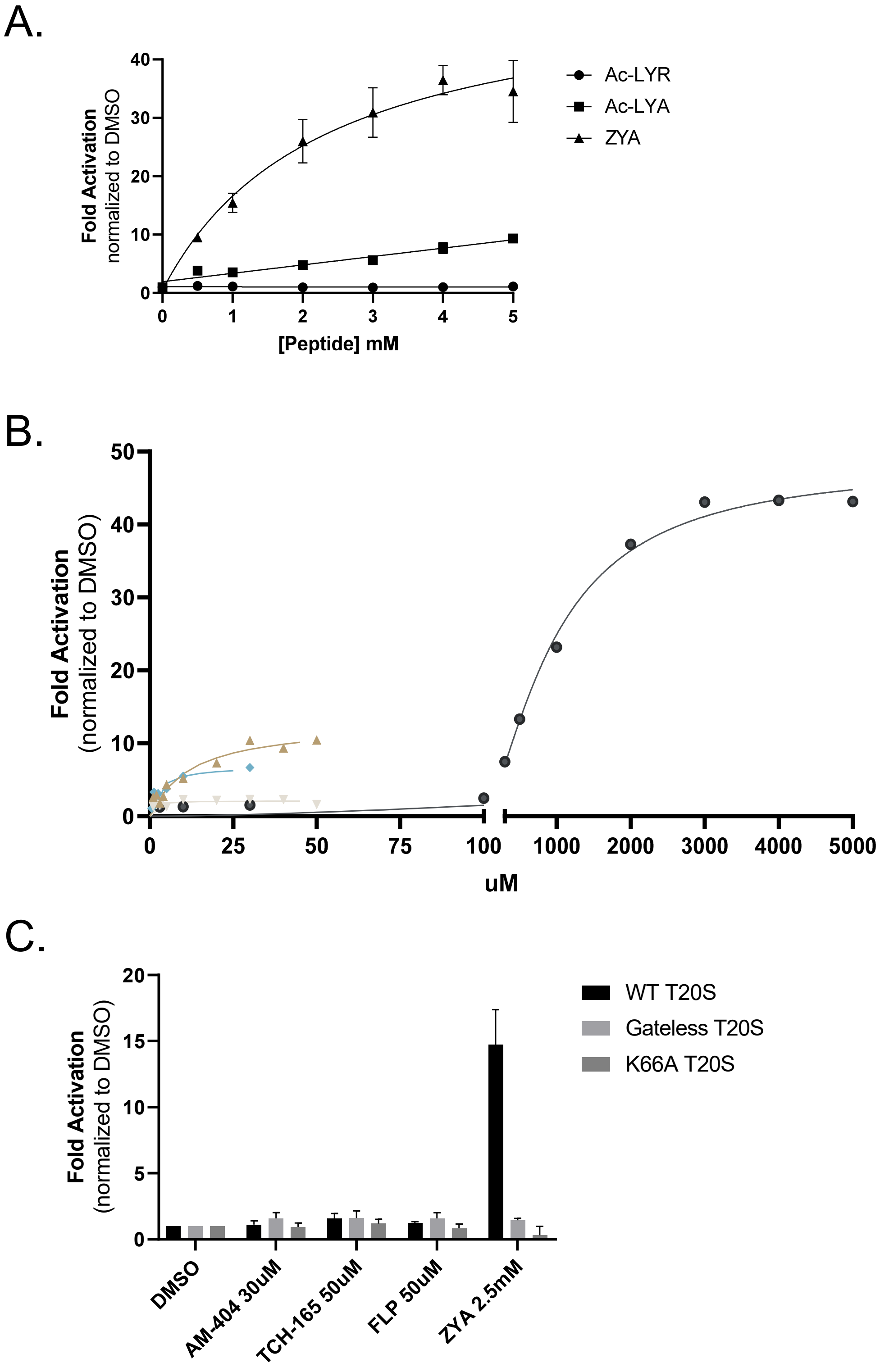

SFig 8: A product of iterative design, ZYA mimics the HbYX motif and outcompetes other published proteasome activators

A. Same as Fig 1J except peptides were incubated at indicated concentrations with mammalian 20S proteasomes (0.5nM) and nLPnLD-amc. Proteasome activity is normalized to DMSO. Experiment performed in triplicate. Error bars represent ± standard deviation.

B. Compounds incubated with mammalian 20S proteasomes (0.5nM) at indicated concentrations and suc-LLVY-amc. Proteasome activity is normalized to DMSO. Fits were generated in Prism using the Michaelis Menten equation for all compounds except ZYA, which was analyzed using the Hill equation. Data are representative of 2-3 independent experiments performed in singlicate dose response.

C. Compounds incubated with 7nM T20S at saturating concentrations as indicated. Proteasome activity was measured by degradation of LFP and normalized to DMSO controls. Data was representative of 3 independent experiments performed in singlicate. Error bars represent ± standard deviation.

Supplementary Files

This is a list of supplementary files associated with this preprint. Click to download.

- SMovie1WT2ZYA.mp4
- SMovie2WT2L81Y.mp4
- SMovie3WT2ZYAGate2.mp4
- SMovie4WT2L81YGate2.mp4
- SMovie5WT21YA7Gate2.mp4
